# Mitochondrial DNA in systemic lupus erythematosus: pathogenic mechanisms, clinical biomarkers, and precision therapeutic strategies

**DOI:** 10.3389/fimmu.2026.1928003

**Published:** 2026-08-13

**Authors:** Fugang Huang, Ke Sun, Lijia Diao, Keda Lu, Yongsheng Fan, Guanqun Xie

**Affiliations:** 1 The First School of Clinical Medicine, Zhejiang Chinese Medical University, Hangzhou, China; 2 The Third School of Clinical Medicine, Zhejiang Chinese Medical University, Hangzhou, Zhejiang, China; 3 The Third Affiliated Hospital of Zhejiang Chinese Medical University (Zhongshan Hospital of Zhejiang Province), Hangzhou, Zhejiang, China; 4 The Second Affiliated Hospital of Zhejiang Chinese Medical University, Hangzhou, China; 5 College of Basic Medical Science, Zhejiang Chinese Medical University, Hangzhou, China

**Keywords:** damage-associated molecular patterns, immune activation, mitochondrial DNA, systemic lupus erythematosus, therapeutic strategy

## Abstract

Mitochondrial DNA (mtDNA) is increasingly recognized as an active driver of immune dysregulation in systemic lupus erythematosus (SLE), yet most existing reviews treat it as a single damage signal rather than a multifunctional pathological mediator. This review presents an integrated framework examining mtDNA as a central hub linking mitochondrial dysfunction to systemic autoimmunity. We conducted a comprehensive synthesis of published evidence on mtDNA biology, its dysregulation in SLE, organ-specific injury mechanisms, and the clinical landscape of mtDNA-targeted therapeutic strategies. In SLE, intracellular mtDNA depletion coexists paradoxically with markedly elevated circulating cell-free mtDNA, a pattern correlating with disease activity and organ involvement. Sequential cytoplasmic and extracellular release of mtDNA engages cyclic GMP-AMP synthase-stimulator of interferon genes (cGAS-STING), Toll-like receptor 9 (TLR9), and inflammasome platforms, establishing self-amplifying interferon and pro-inflammatory circuits that drive multi-organ pathology. We consolidate emerging evidence for mtDNA-related parameters as clinical biomarkers and propose a provisional patient stratification framework distinguishing two pathological subtypes with distinct therapeutic implications. Among current therapeutic strategies, N-acetylcysteine (NAC) and metformin carry the strongest clinical evidence, while cGAS-STING inhibitors and TLR9 antagonists represent compelling emerging candidates. mtDNA operates as an integrative pathological hub in SLE, and its dysregulation pattern carries both diagnostic and therapeutic significance. Incorporating mtDNA-based biomarkers into clinical monitoring and developing precision strategies targeting mtDNA-driven inflammatory circuits represent important steps toward individualized SLE management.

## 1 Introduction

Systemic lupus erythematosus (SLE) is a heterogeneous autoimmune disease characterized by multisystem involvement, disproportionately affecting young women of childbearing age across all ethnic populations (1). Its clinical presentation is remarkably diverse, with manifestations spanning virtually every organ system and varying considerably between patients. The malar “butterfly” rash, a symmetric erythematous eruption across the cheeks and nasal bridge, is among the most recognizable features of the disease. However, SLE extends well beyond this cutaneous hallmark, encompassing polyarthralgia, myalgia, lupus nephritis (LN), cardiovascular disease, and neuropsychiatric involvement. Renal disease is a leading cause of SLE-related morbidity and mortality, typically presenting with proteinuria, microscopic hematuria, and progressive renal dysfunction, while neuropsychiatric manifestations include mood disorders, cognitive impairment, and psychosis (2).

At its pathophysiological core, SLE is driven by the breakdown of immune self-tolerance, leading to autoreactive immune responses. Dysregulated complement activation and the formation and tissue deposition of autoantigen-autoantibody immune complexes sustain systemic inflammation and progressive tissue damage (3). The etiology of SLE is multifactorial, arising from the interplay of genetic susceptibility, environmental triggers, immune dysregulation, and hormonal factors, a combination that makes the disease mechanistically complex and clinically unpredictable (4–6). This underlying complexity renders SLE a chronically relapsing condition that remains difficult to manage, and the identification of upstream molecular drivers capable of simultaneously explaining immune dysregulation, organ-specific injury, and therapeutic resistance remains a central unmet need in the field.

Mitochondrial DNA (mtDNA) has emerged as one such upstream driver, occupying an increasingly prominent position in the mechanistic understanding of SLE. While existing reviews have addressed aspects of mitochondrial dysfunction in autoimmunity, most treat mtDNA principally as a single damage signal acting through discrete innate immune pathways. A more integrated perspective is warranted. In SLE, mtDNA does not simply function as a passive damage-associated molecular pattern (DAMP) released during cellular injury; rather, it operates within a self-amplifying pathological system in which intracellular depletion and extracellular accumulation coexist as mechanistically coupled phenomena, sequential cytoplasmic and extracellular release engages multiple innate immune sensing platforms simultaneously, and the resulting inflammatory output is shaped by mtDNA oxidation status, nucleoid architecture, and cell type-specific sensing capacity. This review synthesizes current evidence for mtDNA as an integrative pathological hub in SLE, examines the mechanistic basis for its organ-specific injury patterns, evaluates the clinical evidence for mtDNA-targeted therapeutic strategies, and proposes a framework for mtDNA-based patient stratification that may inform future precision therapeutic approaches in SLE.

## 2 MtDNA: biological properties and functional relevance to immune activation

### 2.1 Evolutionary origins and maternal inheritance of mtDNA

Mitochondrial DNA (mtDNA) is a multi-copy, circular double-stranded molecule of approximately 16,500 base pairs that resides in the mitochondrial matrix. It encodes 37 genes: 13 protein-coding genes essential for the respiratory chain and ATP synthesis, 22 transfer RNA genes, and 2 ribosomal RNA genes. Together, these genes sustain mitochondrial energy production and cellular homeostasis within an autonomous genetic system embedded in the mitochondrion itself (7, 8).

mtDNA inheritance is strictly maternal: the oocyte supplies the entire mitochondrial complement, while sperm contribute only nuclear DNA. As a result, mtDNA is transmitted through the matrilineal lineage with negligible paternal contribution. Furthermore, mtDNA undergoes virtually no recombination during cell division, preserving genomic configurations that closely resemble ancestral templates, a feature underlying its well-established utility in phylogenetic reconstruction (9, 10).

The conservation of mtDNA sequences has made it an invaluable tool for tracing maternal lineages and understanding hereditary mitochondrial disorders such as Leber’s hereditary optic neuropathy (11) and mitochondrial myopathy (12). Critically, because the genomic architecture of mtDNA diverges substantially from that of nuclear DNA in both structure and transcriptional regulation, aberrant immune recognition of mtDNA can trigger potent and pathological innate immune activation, a property that is central to its role in SLE pathogenesis.

### 2.2 High mutability of mtDNA and its immunological consequences

Compared with nuclear DNA, mtDNA has a substantially higher mutation rate, attributable to both its structural features and its intracellular environment (13). As an unprotected circular molecule directly exposed within the mitochondrial matrix, mtDNA lacks the histone-based chromatin shielding that guards nuclear DNA (14).Because mitochondria are the principal intracellular source of reactive oxygen species (ROS), mtDNA is subject to persistent oxidative stress. ROS produced during electron transport chain (ETC) activity directly damage mtDNA, and oxidatively modified mtDNA displays enhanced immunogenicity that further propagates pro-inflammatory signaling (7).

The compact genomic organization of mtDNA, with few introns and minimal non-coding sequences, means that the majority of mutations directly impair coding regions. This disrupts key subunits of respiratory chain complexes I, III, IV, and V, as well as the tRNAs and rRNAs required for mitochondrial translation. The resulting chain of consequences, encompassing respiratory insufficiency, reduced ATP output, increased ROS production, and further mtDNA deterioration, forms a self-amplifying cycle of cellular injury (15, 16). The temporal asymmetry inherent in bidirectional mtDNA replication, in which heavy strand synthesis lags behind, further prolongs single-stranded DNA exposure and increases mutational susceptibility (17).

Clinically, mtDNA mutations underlie a range of primary mitochondrial diseases, including Leber hereditary optic neuropathy (LHON) (11), mitochondrial encephalomyopathy with lactic acidosis and stroke-like episodes (MELAS) (18), and myoclonic epilepsy with ragged red fibers (MERRF) (19), conditions that disproportionately affect tissues with high bioenergetic demands such as the central nervous system, heart, and skeletal muscle. In the context of autoimmunity, the relevance of mtDNA mutability lies not only in its functional consequences for cellular metabolism but also in its capacity to generate structurally altered DNA species with heightened immunostimulatory potential, as discussed in subsequent sections.

### 2.3 Nucleoid-like architecture of the mtDNA-TFAM complex

mtDNA assembles with mitochondrial transcription factor A (TFAM) and a set of accessory proteins, including DNA polymerase γ (Polγ), mitochondrial single-stranded DNA-binding protein (mtSSB), transcription factors TFB1M/TFB2M, and mitochondrial RNA polymerase (POLRMT), to form nucleoid structures that constitute the fundamental organizational unit of the mitochondrial genome (20, 21). TFAM serves as the primary structural scaffold, binding mtDNA through its HMG-box domains and bending the DNA to compact the circular genome into dense granular particles (22–25). The functional significance of this architecture for SLE lies in two properties in particular: the compacted nucleoid physically shields mtDNA from ROS-mediated oxidative damage (26, 27), and TFAM binds preferentially to damaged mtDNA and participates directly in base excision repair (28). Nucleoid integrity therefore determines both how readily mtDNA is oxidized and how efficiently oxidative lesions are corrected.

Nucleoid assembly is coupled to cellular energy status. Import of TFAM into the mitochondrial matrix is rate-limiting for nucleoid formation (29), and TFAM is transcriptionally upregulated downstream of PGC-1α when bioenergetic demand rises, enhancing expression of mitochondrially encoded respiratory chain subunits (30, 31). Nucleoid density accordingly varies with tissue metabolic activity and increases under conditions of elevated energy demand (25, 32, 33).

Nucleoid integrity is maintained through tight regulatory control of its disassembly. Aberrant activation of Lon protease accelerates TFAM proteolysis and triggers nucleoid dissolution (34), while GCN5L1-mediated acetylation of TFAM at K76 suppresses its mitochondrial import and functional activity (35). Deficiency or dysfunction of TFAM leads to nucleoid structural abnormalities and quantitative depletion, directly impairing mtDNA stability and transcription (29, 36, 37); in mice, embryonic TFAM deletion is lethal and tissue-specific disruption causes severe early disease (38).

Nucleoid destabilization has been documented in a range of conditions outside autoimmunity, including MELAS, Parkinson’s disease, Alzheimer’s disease, aging, and diabetes, in which pathogenic mtDNA mutations, protein aggregates competing with TFAM for DNA binding, or cumulative oxidative and glycative modification of TFAM converge on the same outcome of nucleoid loosening and mtDNA depletion (39–49). Nucleoid dysfunction can additionally influence nuclear gene expression through retrograde AMPK-PGC-1α signaling, though this compensatory response does not fully offset the metabolic consequences of nucleoid disruption (50–52). These observations establish that nucleoid integrity is a general determinant of mitochondrial genomic stability.

### 2.4 From life-sustaining code to damage signal: cellular consequences of mtDNA leakage

Given its bacterial evolutionary origin, high gene density, and unique codon usage, mtDNA is maintained under a tightly regulated balance between replication and degradation (53, 54). mtDNA replication initiates at the heavy strand origin within the D-loop and proceeds by asymmetric strand displacement driven by Polγ, the sole mitochondrial DNA polymerase, which carries intrinsic 3’→5’ exonuclease proofreading activity. The helicase Twinkle unwinds the duplex at the replication fork, while mtSSB stabilizes exposed single-stranded DNA (53, 54). Light strand replication initiates with a characteristic delay relative to the heavy strand (55), though a competing strand-coupled bidirectional model proposes greater temporal coordination between strand syntheses and the question remains unresolved (56). Replication is coupled to cellular energy status through the AMPK-PGC-1α axis, and mtDNA copy number is correspondingly tissue-specific, ranging from thousands of copies in cardiomyocytes to hundreds in fibroblasts, and declines with aging alongside accumulating mutational burden (25).

Quality control relies on a multilayered set of repair pathways with distinct substrate specificities. Base excision repair is the primary and best-characterized mechanism and is particularly efficient against oxidative lesions (57, 58). Oxidative adducts such as 8-oxoguanine and thymine glycol are excised by the glycosylases OGG1 and NEIL1, followed by gap-filling synthesis involving APE1, Polγ, and LIGIII (59, 60). MGME1 processes RNA primers and damaged fragments generated during replication (61), and a mitochondrial mismatch repair system distinct from its nuclear counterpart has also been described (62). Homologous recombination and non-homologous end joining address double-strand breaks (63, 64). Despite this repertoire, overall repair capacity is limited: nucleotide excision repair is absent, Polγ proofreading is constrained, and repair kinetics under sustained oxidative stress lag substantially behind those of nuclear DNA (65–67). The resulting mutational landscape encompasses point mutations, large-scale deletions, and D-loop mutations that alter replication and transcription efficiency despite lacking coding capacity (68–71).

When repair capacity is exceeded, damaged mtDNA is eliminated by intramitochondrial nucleolytic cleavage, mediated principally by endonuclease G with contributions from ATAD3B and TREX1 (72–75), or by mitophagy. In the latter, nucleoid disassembly converts TFAM from a structural guardian into an autophagosomal adaptor that directs damaged mtDNA into autophagolysosomes for DNase II degradation (75). Impaired mitophagy has been shown to permit accumulation of damaged mitochondria in Parkinson’s disease, Alzheimer’s disease, and diabetes, through loss-of-function mutations in PINK1 and Parkin, inhibition of PINK1 by Aβ oligomers, and hyperglycemia-driven suppression of autophagic flux respectively (76–80). These findings define the general consequences of mitophagic failure but derive from non-autoimmune contexts.

When clearance fails, leaked mtDNA initiates damage across several interconnected levels. As a DAMP, cytosolic mtDNA is sensed by intracellular pattern recognition receptors (PRRs): TLR9 recognizes CpG-rich sequences and activates NF-κB and AP-1 via MyD88, cGAS binds mtDNA and synthesizes cGAMP to activate STING and trigger type I interferon responses, and AIM2 nucleates an inflammasome with ASC and Caspase-1 to drive IL-1β and IL-18 maturation (81–83). Leaked mtDNA additionally engages multiple cell death programs, including intrinsic apoptosis through Bax and Bak pore formation, necroptosis, pyroptosis, and ferroptosis, modalities that frequently interact to accelerate cell loss (84–86). Oxidative damage is both cause and consequence: defective respiratory chain assembly generates excess ROS causing protein carbonylation, phospholipid peroxidation, and oxidative base modification (87–90). At the metabolic level, impaired oxidative phosphorylation reduces ATP output and drives compensatory glycolysis with lactate accumulation, while fatty acid β-oxidation, TCA cycle flux, and one-carbon metabolism are all disrupted (91–95). These processes amplify one another, establishing a self-reinforcing cycle of cellular dysfunction.

## 3 Multidimensional dynamics of mtDNA in SLE pathogenesis

Throughout the pathogenesis of SLE, mtDNA undergoes a spectrum of aberrant alterations that not only reflect the underlying disease state but actively participate in its progression and organ-specific injury, functioning simultaneously as a biomarker and a mechanistic driver of immunopathology.

### 3.1 Bidirectional dysregulation: the paradoxical coexistence of intracellular depletion and extracellular accumulation

Within the pathological landscape of SLE, mtDNA homeostasis is disrupted in a distinctive and mechanistically revealing pattern that involves simultaneous quantitative changes across intracellular and extracellular compartments. At the population level, SLE patients exhibit an ostensibly paradoxical yet functionally interconnected biphasic alteration: mtDNA copy numbers within peripheral blood mononuclear cells (PBMCs) are significantly reduced, a reduction that correlates with established disease activity indices and tracks disease trajectory over time (96–98), while circulating cell-free mtDNA is concurrently elevated, likewise concordant with SLEDAI scores (99). This pattern of intracellular depletion coexisting with extracellular accumulation reflects a fundamental breakdown in mtDNA homeostasis, wherein compromised intracellular biosynthesis or accelerated degradation converges with pathological release driven by mitochondrial membrane disruption, programmed cell death, or cellular necrosis. The direction of this relationship, however, remains undetermined. Cross-sectional cohorts cannot establish whether intracellular depletion precedes immune activation or arises as its consequence, and longitudinal sampling across flare and remission has not been reported.

Intracellular mtDNA depletion is invariably accompanied by qualitative deterioration of the residual genome. Through the strategic deployment of long- and short-fragment amplification assays, Ayala-Peña et al. demonstrated that amplification of extended mtDNA fragments from SLE patient PBMCs is markedly impaired, a consequence of replicative polymerase stalling at oxidative lesion sites, while short-fragment amplification, which reflects aggregate copy number, also declines independently (97). This molecular phenotype indicates that intracellular mtDNA contraction in SLE encompasses both oxidative functional impairment and comprehensive defects in mtDNA replication and maintenance machinery. Of particular significance, this pattern of mtDNA disruption is present across SLE patients irrespective of organ involvement status, suggesting that it constitutes an intrinsic pathological feature of the disease rather than a secondary epiphenomenon. Oxidative stress occupies a central mechanistic role in this process; peroxynitrite (ONOO^−^)-mediated modification of mtDNA not only inflicts direct structural damage but also generates structurally altered neo-epitopes, potentially converting modified mtDNA into an endogenous autoantigen capable of perpetuating self-reactive immune responses (100).

In striking contrast to its intracellular depletion, mtDNA accumulates markedly in the extracellular compartment of SLE patients, establishing it as a potent DAMP and a critical molecular nexus linking mitochondrial dysfunction to innate immune activation (101). Investigations examining immune reactivity profiles in SLE patient sera reveal that immunoreactivity toward mtDNA substantially surpasses that directed against nuclear DNA, a selectivity that likely reflects the bacterial evolutionary ancestry of mtDNA and its consequent enrichment in unmethylated CpG motifs, which confer high recognition efficiency by pattern recognition receptors (102). Beyond mtDNA itself, nucleoid structures represent an additional source of DAMPs in SLE pathology. Anti-TFAM autoantibodies detected in SLE sera exhibit a clinically distinctive profile that sets them apart from conventional anti-dsDNA antibodies: rather than correlating with disease activity metrics or interferon signatures, these antibodies show robust associations with thrombotic manifestations, antiphospholipid syndrome, thrombosis-related transcriptional signatures, and interferon-λ elevation, an association that extends to primary antiphospholipid syndrome, suggesting that anti-TFAM antibodies define a discrete immunopathological subset with a specific contribution to SLE-associated thrombogenesis (103).

The immunogenic potential of mtDNA extends well beyond the DNA molecule itself. SLE patient sera contain autoantibodies against mitochondrial RNA (mtRNA), with significant elevation of both IgG and IgM isotypes. Anti-mtRNA IgG titers correlate positively with anti-mtDNA IgG and anti-β2-glycoprotein I antibody levels while demonstrating inverse correlations with atherosclerotic plaque formation and LN incidence; Anti-mtRNA IgM, by contrast, shows minimal clinical associations (104). The immunogenicity of mtRNA is mechanistically rooted in defects in mitochondrial quality surveillance: polynucleotide phosphorylase (PNPase), resident in the mitochondrial intermembrane space, normally degrades mtRNA species harboring pathogen-mimetic structural motifs, preventing their cytoplasmic escape as DAMPs. Patients harboring PNPT1 mutations show abnormal accumulation of mitochondrial double-stranded RNA (ds-mtRNA) that correlates with transcriptional upregulation of type I interferon-stimulated genes and pro-inflammatory mediators (105). Cytoplasmic ds-mtRNA is recognized by protein kinase R (PKR); the resulting PKR-ds-mtRNA complex not only directly triggers type I interferon secretion but also modulates translational efficiency through eIF2α phosphorylation, thereby establishing a pathway that directly couples mitochondrial quality control failure to systemic inflammatory amplification (106). Additionally, anti-mitochondrial M2 antibodies are elevated in subacute cutaneous lupus erythematosus (107), while elevated anti-whole mitochondria antibody (anti-wMITO) titers correlate with SLEDAI-2K, SLAM scores, and anti-dsDNA antibodies (108), collectively affirming the broad immunogenic involvement of diverse mitochondrial antigens in SLE pathophysiology.

Genetic variation in the mitochondrial genome shapes the mtDNA disruption profile in SLE, and the reported associations fall into three broad categories that differ in both mechanism and evidential strength. The first concerns inherited population-level background. Phylogenetic analysis of mtDNA M/N haplogroups indicates that N haplogroup carriers are more susceptible to hematological, cutaneous, and neurological manifestations as well as alopecia (109), suggesting that ancestral mitochondrial lineage may influence which organ systems are affected rather than disease risk alone.

The second comprises variants in coding and regulatory regions, most identified through case-control association studies. Whole mitochondrial genome sequencing has flagged four candidate disease-associated variants, of which A4833G and G14569A were associated with elevated relapse risk (110). Within the D-loop regulatory region, polymorphisms at positions 73, 195, and 199 have been linked to SLE predisposition (111), and leukocyte D310 heteroplasmy frequency correlates positively with SLEDAI scores (112). Several single nucleotide polymorphisms show phenotype-specific associations, with nt16189C linked to overall susceptibility, nt13708A to male-onset disease, and nt10398A to secondary antiphospholipid syndrome (113); variants in UCP2 and ATP6 have likewise been reported (114, 115). These associations are largely statistical, derived from cohorts of modest size, and have not been consistently replicated across populations.

**Figure 1:**
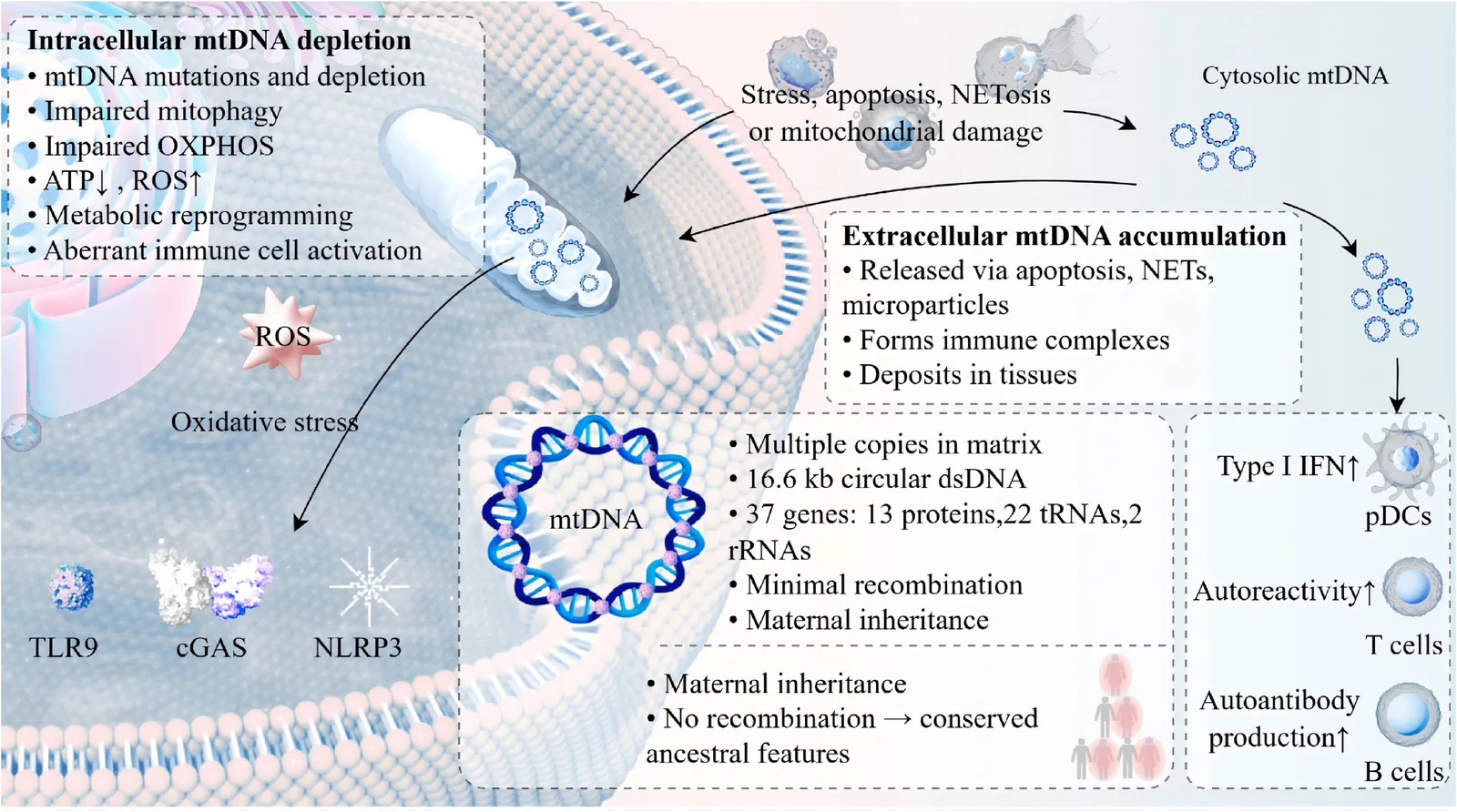
Bidirectional dysregulation of mtDNA in SLE: from intracellular depletion to extracellular immune activation. Mitochondrial DNA (mtDNA) is a maternally inherited, 16.6 kb circular double-stranded DNA molecule encoding 37 genes, including 13 protein-coding genes, 22 tRNA genes, and 2 rRNA genes. In SLE, mtDNA homeostasis is disrupted in a paradoxical pattern: intracellular mtDNA undergoes depletion driven by mutations, impaired mitophagy, oxidative phosphorylation (OXPHOS) failure, and elevated reactive oxygen species (ROS), leading to metabolic reprogramming and aberrant immune cell activation. Concurrently, cellular stress, apoptosis, NETosis, and mitochondrial damage drive mtDNA release into the cytosol and extracellular space. Extracellular mtDNA accumulates in the form of immune complexes and tissue deposits, released through apoptosis, neutrophil extracellular traps (NETs), and microparticles. Cytosolic mtDNA activates innate immune sensors including TLR9, cGAS, and NLRP3, triggering type I interferon production, T cell autoreactivity, and B cell survival and autoantibody generation, collectively amplifying the systemic inflammatory response characteristic of SLE.

The third category, mutations affecting mitochondrial translation, carries the most direct functional evidence. A sequencing survey of 200 SLE patients identified five candidate pathogenic mt-tRNA variants that were detected exclusively in patients and localized to highly conserved tRNA positions, where they disrupt secondary structure (116). Cells carrying these variants show abnormalities in ATP levels, mtDNA copy number, mitochondrial membrane potential, and oxidative stress parameters, which together implicate translational insufficiency as a plausible contributory mechanism. Even here, however, the functional data derive from cellular models rather than from patient tissue, and whether these variants are causal or represent markers of a broader mitochondrial phenotype remains undetermined (**Figure 1**).

#### 3.1.1 mtDNA-based biomarkers in SLE: toward an integrated clinical framework

The convergence of mechanistic and clinical evidence positions mtDNA-related parameters as a particularly promising category of biomarkers in SLE, one that may complement or one that may complement established serological markers in specific clinical contexts. The utility of mtDNA as a biomarker derives directly from the bidirectional dysregulation described above: intracellular mtDNA copy number reflects the integrity of mitochondrial maintenance pathways, while circulating cell-free mtDNA and its associated molecular signatures capture the degree of mitochondrial damage and immune activation at the systemic level.

Several distinct mtDNA-related parameters have been evaluated in clinical cohorts, each with a different biomarker profile. Circulating cell-free mtDNA concentration is the most extensively studied parameter, with multiple studies demonstrating significant elevation in SLE patients compared with healthy controls and patients with other autoimmune diseases, and positive correlation with SLEDAI scores and renal involvement (96–99). Importantly, circulating cell-free DNA (cfDNA) profiling that incorporates fragment size distribution, specifically the presence of 54–149 bp and 209–297 bp fragments alongside absolute concentration and the intracellular-to-extracellular mtDNA copy number ratio, provides substantially greater discriminative power than concentration alone, stratifying patients according to renal functional trajectory and risk of progressive lupus nephritis (117). Oxidative damage indices, most notably 8-OHdG modification burden in leukocyte mtDNA, correlate with SLEDAI scores and anti-dsDNA titers, suggesting their potential utility as markers of disease activity and cumulative oxidative stress (118, 119). Anti-mtDNA antibodies represent a third biomarker category, demonstrating stronger associations with lupus nephritis severity than conventional anti-dsDNA titers and showing particular utility in identifying patients at risk of pDC-driven interferon amplification through TLR-mediated immune complex signaling (120–122). Anti-TFAM antibodies define a clinically distinct subset characterized by thrombotic risk and antiphospholipid syndrome associations rather than inflammatory disease activity, suggesting their value in identifying patients who may benefit from anticoagulant rather than immunosuppressive intensification (103). mtDNA mutation burden, including D-loop D310 heteroplasmy frequency and the presence of specific tRNA variants, may further inform long-term prognostication, given their associations with renal functional impairment and disease relapse risk (110, 112).

Integrating these parameters into a coherent clinical framework requires recognition that different mtDNA biomarkers capture distinct pathophysiological processes and therefore carry different clinical utilities. On the basis of available evidence, we propose a provisional stratified approach in which circulating cfDNA profiling and oxidative damage indices serve as dynamic markers of disease activity and organ involvement (96, 98, 99, 119, 120), anti-mtDNA and anti-TFAM antibodies inform subtype classification and risk stratification for specific complications (102, 121, 123), and mtDNA mutation burden provides prognostic information regarding long-term disease trajectory (110, 112, 116). This framework is summarized in **Table 1**.

**Table 1:** mtDNA-related biomarkers in SLE: parameters, clinical associations, and proposed utility.

Biomarker parameter	Clinical associations	Proposed clinical utility	Current limitations
Circulating cell-free mtDNA concentration	Correlates with SLEDAI, renal involvement	Disease activity monitoring	Lacks specificity; affected by non-SLE conditions
cfDNA molecular profile (fragment size, copy number ratio)	Predicts eGFR trajectory and LN progression risk	Renal risk stratification	Requires standardized multi-parameter assay
Leukocyte mtDNA 8-OHdG burden	Correlates with SLEDAI and anti-dsDNA titers	Oxidative stress monitoring	Limited prospective validation
Anti-mtDNA antibodies (IgG)	Associates with LN severity, IFN signatures	LN severity assessment; pDC activation risk	Assay not widely standardized
Anti-TFAM antibodies	Associates with thrombosis, antiphospholipid syndrome, IFN-λ	Thrombotic risk stratification	Novel; requires prospective validation
D-loop D310 heteroplasmy	Correlates with SLEDAI, renal involvement	Long-term prognostication	Research tool; clinical translation pending
mt-tRNA pathogenic variants	Associated with relapse risk, metabolic impairment	Disease susceptibility and relapse risk	Limited cohort sizes

Translation of this framework nonetheless faces substantial obstacles. No mtDNA-related parameter has undergone prospective validation, assay standardization, or head-to-head comparison against established serological markers, and circulating cell-free mtDNA is not disease-specific, rising also in sepsis, trauma, and malignancy. The realistic near-term role for these parameters is as exploratory endpoints within clinical trials rather than as tools for individual patient management.

### 3.2 the sequential liberation of mtDNA: from cytoplasm to extracellular space

mtDNA release in SLE is a dynamically regulated, multistage process that proceeds through two operationally discrete yet mechanistically coupled phases: intracytoplasmic translocation and extracellular extrusion. Under normal physiological conditions, mtDNA is spatially confined within the mitochondrial double-membrane system and functionally protected by an elaborate network of quality control mechanisms. In the SLE pathological environment, this homeostatic equilibrium is systematically disrupted, and mtDNA traverses successive compartments through cascading cytoplasmic redistribution and extracellular dissemination, establishing a self-amplifying inflammatory circuit that lies at the mechanistic core of disease perpetuation.

#### 3.2.1 Intracytoplasmic release of mitochondrial DNA

The aberrant cytosolic redistribution of mtDNA represents a central pathological event implicated in both disease initiation and autonomous amplification within the mechanistic framework of SLE. Three interconnected processes underlie this redistribution: maladaptive remodeling of mitochondrial ultrastructure, which directly compromises the spatial anchoring of mtDNA nucleoid complexes; progressive dissolution of membrane integrity, which establishes physical conduits for trans-membrane mtDNA egress; and failure of quality surveillance networks, which allows oxidatively damaged mtDNA to evade degradative elimination and accumulate at pathological concentrations. Operating within the SLE immune microenvironment, these converging mechanisms culminate in dysregulated cytoplasmic mtDNA release that engages innate immune machinery with profound downstream consequences.

In the predominant effector cell populations of SLE patients, including activated T cell subsets comprising naïve (Tn), effector memory (Tem), and central memory (Tcm) cells, plasmacytoid dendritic cells (pDCs), and follicular helper T cells (Tfh), mitochondria exhibit pronounced morphological and functional abnormalities that directly compromise nucleoid organizational coherence and spatial anchoring fidelity, with the degree of these alterations correlating robustly with clinical disease activity indices (124, 125).

Mitochondrial matrix swelling, a characteristic feature of dysfunctional mitochondria in SLE, dilutes intramatrix protein concentrations and thereby attenuates TFAM’s capacity to package and structurally protect mtDNA (126). More critically, the mechanical forces generated by matrix volumetric expansion act directly upon the attachment sites that tether mtDNA to the inner membrane, interactions mediated primarily through protein-protein contacts between the ATAD3 complex and inner membrane-associated proteins. As mechanical stress escalates beyond a critical threshold, these molecular tethers dissociate, releasing nucleoids from their inner membrane scaffold, conferring enhanced diffusional mobility within the matrix, and establishing the prerequisite conditions for subsequent trans-membrane translocation (74, 127, 128).

Mitochondrial cristae, the elaborately folded inner membrane invaginations that project into the matrix, undergo progressive fragmentation, regional ablation, and topological collapse across multiple SLE effector cell populations (129, 130). The integrity of cristae architecture has dual implications for mtDNA spatial organization: first, cardiolipin-enriched microdomains within cristae membranes serve as preferred nucleoid anchoring sites, maintained through electrostatic interactions between negatively charged cardiolipin headgroups and cationic domains of TFAM; second, the topological architecture of cristae physically constrains mtDNA diffusion to circumscribed intramatrix regions (131). Cristae structural disruption thus eliminates preferred nucleoid anchoring sites while simultaneously removing the topological barriers that normally restrict mtDNA mobility, expanding the diffusional range of nucleoids and substantially increasing their probability of approaching the outer membrane interface (131). Notably, the resulting redistribution of mtDNA nucleoids within T cells from SLE patients is sensed by ENPP1, which amplifies GLUT1 expression and glycolytic flux while suppressing lipogenic pathways; concurrently, impaired N-myristoylation and lysosomal trafficking of AMPK lead to its functional inactivation, subsequent mTOR activation, and aberrant effector T cell differentiation (132).

The oxidative microenvironment characteristic of SLE inflicts direct biochemical damage upon mtDNA nucleoid architecture at multiple levels. mtDNA 8-OHdG modification burden is significantly elevated in SLE patients relative to healthy controls, with levels correlating robustly with SLEDAI scores and anti-dsDNA antibody titers (118, 119). Dysregulated ROS overproduction exerts dual oxidative insults upon both mtDNA and TFAM: oxidative base lesions destabilize the double helical architecture of mtDNA, rendering it susceptible to TFAM dissociation and nucleoid unpackaging (103, 133); simultaneously, aberrant ROS accumulation drives oxidative post-translational modification of TFAM, particularly methionine-to-methionine sulfoxide conversion, while upregulated Lonp1 protease activity promotes selective TFAM proteolysis, both mechanisms converging to reduce TFAM’s mtDNA-binding affinity and accelerate nucleoid dissolution (29, 34, 134). The resulting naked, unshielded mtDNA, with its exposed unmethylated CpG motifs and double-stranded DNA backbone, constitutes a highly potent ligand for cytosolic DNA sensing machinery upon cytoplasmic release.

The shift in mitochondrial dynamics toward excessive fragmentation that characterizes SLE effector cells further facilitates cytoplasmic mtDNA release (124, 135, 136). An imbalance between Drp1-mediated hyperfission and reduced expression of the fusion mediators Mfn1/2 and OPA1 generates abundant small, fragmented mitochondria that, by virtue of their elevated surface-to-volume ratios and lack of network-buffering capacity, are substantially more vulnerable to localized membrane perturbations. Critically, the reduced nucleoid complement per fragmented mitochondrion means that individual nucleoids are, on average, positioned in closer proximity to the outer membrane, such that localized outer membrane rupture or mPTP opening substantially increases the probability of non-programmed mtDNA cytoplasmic egress. Real-time fluorescence imaging has confirmed that following oxidative stress provocation, mtDNA release kinetics from hyper-fragmented mitochondria significantly exceed those from intact reticular mitochondrial networks (137, 138).

The mitochondrial permeability transition pore (mPTP), formed through pathological sustained VDAC oligomerization, provides the principal structural conduit for trans-membrane mtDNA translocation. mtDNA interacts with three cationic residues (Lys12, Arg15, Lys20) at the VDAC1 N-terminus, enabling extrusion through the oligomeric pore assembly and representing the critical step that converts potential cytoplasmic translocation into actuality (139, 140). VDAC1 and VDAC3 are overexpressed in SLE (141), and moderate mitochondrial stress is sufficient to promote mPTP-dependent mitochondrial outer membrane permeabilization (MOMP) and mtDNA release independently of BAX/BAK macropore formation, culminating in lupus-like phenotypic manifestations (140). Under SLE pathophysiological conditions, the frequency of mPTP opening events is dramatically elevated compared with healthy cells, transitioning from transient openings that release small ions and metabolites to sustained openings that permit macromolecular permeation including nucleic acids (140); sustained aperture can additionally cause secondary outer membrane rupture, creating enlarged channels for mtDNA egress.

The SLE immune microenvironment is thought to promote dysregulated mPTP opening through several interconnected mechanisms. ROS generated by type I interferon signaling drives oxidative modification of Cyclophilin D (CypD), lowering the calcium threshold for mPTP opening, while concurrently causing oxidative mtDNA damage that promotes nucleoid dissociation and diffusion toward pore regions (142, 143). Pathologically elevated mitochondrial matrix calcium, a feature of dysregulated calcium homeostasis in SLE, independently can trigger mPTP opening and, through activation of matrix calcium-dependent endonucleases, fragments mtDNA into smaller species that more readily traverse pores or outer membrane breaches (144). Matrix osmotic swelling resulting from sustained mPTP activity promotes mtDNA release through two additional mechanisms: pressure gradients generated by volumetric expansion actively propel nucleoids toward pore regions, while extreme swelling can cause inner membrane herniation sufficient to rupture the outer membrane, creating micrometer-scale apertures through which intact nucleoids or large-fragment mtDNA can transit directly into the cytoplasm (145, 146).

Mitophagy is broadly suppressed in SLE patients, allowing dysfunctional mitochondria harboring substantial quantities of oxidatively modified mtDNA to evade degradative clearance and accumulate within the cytoplasm, establishing a sustained reservoir for ongoing mtDNA release (124, 147–149). Within this pathological context, type I interferon signaling suppresses autophagic activity through ISG15ylation modifications (147, 150) and impairs autophagic flux via lysosomal alkalinization (151), collectively preventing timely clearance of damaged mitochondria in immune cells. These retained dysfunctional mitochondria continuously generate ROS and release mtDNA, which would be expected to drive progressive oxidative damage and may selectively enrich the most immunogenically potent mtDNA species. Concomitantly, autophagosome-lysosome fusion efficiency is reduced (152, 153), such that mitochondria sequestered within stalled autophagosomes face two pathological fates: cytoplasmic re-release of mtDNA through enhanced autophagosomal membrane permeability, or partial hydrolysis by the acidic intraluminal environment that generates small-fragment mtDNA with enhanced diffusional mobility and intercellular trafficking capacity (151). The progressive accumulation of such stalled autophagosomes, well-documented throughout SLE disease progression (154), may create a reservoir amplification effect in which the growing pool of damaged mitochondria and increasing outer membrane fragility contribute to the elevated cytoplasmic mtDNA concentrations measured in SLE patient cohorts (96, 98, 99).

Released mtDNA, bearing molecular signatures inherited from its bacterial endosymbiotic ancestry, activates multiple PRRs of the innate immune system, initiating pro-inflammatory signaling cascades that in turn amplify further mtDNA liberation and modification. Recognition by cGAS is strongly influenced by the oxidation status, fragment length, and conformation of mtDNA (144). Under the elevated oxidation states characteristic of the SLE cytoplasmic environment, released mtDNA appears to constitute a particularly efficient cGAS substrate: 8-oxoguanine-induced conformational distortion increases interfacial contact with the cGAS catalytic site, while oxidative modifications that weaken TFAM-mtDNA binding expose additional phosphodiester backbone for sensor engagement (103, 133). Downstream activation generates 2’3’-cGAMP, which engages STING to induce IFN-α/β and pro-inflammatory cytokine expression (155). In SLE monocytes, cGAS expression is markedly upregulated and its binding kinetics for mtDNA are altered, consistent with a self-sustaining activation circuit (156, 157).

This circuit is reinforced from the interferon side. IFN-α produced by mtDNA-stimulated pDCs promotes further mtDNA release through several routes, upregulating NOX2 to increase ROS generation, suppressing autophagy through ISG15ylation, and downregulating antioxidant enzymes, thereby maintaining cytoplasmic mtDNA in a persistently oxidized state (151, 156). Notably, cGAS-STING activation shows a non-linear relationship with cytoplasmic mtDNA concentration, in which STING oligomerization efficiency rises sharply beyond a threshold (158); SLE patient mtDNA concentrations may fall within this amplification range, which would allow modest fluctuations in mtDNA to produce disproportionate changes in interferon output (151, 159). Whether this dynamic contributes to the episodic nature of lupus activity remains speculative but is experimentally testable. A TOP1MT variant (P193L) identified in a lupus kindred offers indirect support for the broader premise: TOP1MT is the only human topoisomerase confined to mitochondria (160), and functional impairment is thought to compromise mtDNA maintenance and permit aberrant cytoplasmic release (161).

TLR9, predominantly expressed within intracellular endosomal and lysosomal compartments, recognizes unmethylated CpG dinucleotides in mtDNA, and binding of released mtDNA to TLR9 engages the TLR9-MyD88-IRF7 signaling axis to drive IFN-α production, a principal contributor to SLE-associated hyperinterferonemia (162–164). Notably, TLR9 activation is not solely dependent on CpG methylation status but is additionally potentiated by oxidative modification of mtDNA, as oxidized CpG dinucleotides show enhanced TLR9-activating capacity relative to unmodified counterparts, possibly through altered DNA-TLR9 binding geometry (165–167); the elevated oxidation burden and supraphysiological concentrations of circulating mtDNA in SLE patients thus generate converging activation signals that synergistically drive excessive TLR9 pathway engagement.

NLRP3 inflammasome sensitivity is further enhanced by oxidative mtDNA modification, as oxidized bases may directly engage the leucine-rich repeat (LRR) domain of NLRP3 to facilitate conformational change and oligomerization (168, 169), while ROS generated by oxidized mtDNA provides a canonical second signal for NLRP3 activation, establishing a dual-hit mechanism in which mtDNA simultaneously supplies both priming and activating stimuli (170, 171). NLRP3-driven IL-1β maturation amplifies the inflammatory cascade, and AIM2 inflammasome recognition of cytosolic mtDNA further contributes through Caspase-1 activation and additional IL-1β secretion; during these processes, IL-1β promotes mitochondrial fragmentation via Drp1 upregulation, increasing mtDNA release efficiency and closing another positive feedback loop (172–174). The causal role of IL-1β in this circuit is supported by the robust positive correlation between serum IL-1β concentrations and cytoplasmic mtDNA levels in SLE patients, and by the observation that IL-1β antagonist treatment reduces cytoplasmic mtDNA burden (159, 175).

Downstream of inflammasome activation, gasdermin D (GSDMD) represents a mechanistically distinct node at which oxidized mtDNA acts. Conventionally, GSDMD is released from autoinhibition by caspase-1 cleavage, and the resulting N-terminal fragment oligomerizes to form membrane pores that permit IL-1β efflux. In lupus, however, oxidized mtDNA appears to engage GSDMD directly: Miao and colleagues reported that oxidized mtDNA binds the GSDMD N-terminal domain and promotes its oligomerization, that oxidized mtDNA is enriched in SLE neutrophils and monocytes relative to healthy controls, and that GSDMD oligomerization correlates with disease activity (176). Oxidized mtDNA therefore acts not only as an upstream ligand priming inflammasome assembly, but also as a direct effector at the terminal step of pore formation. The consequence is a circuit rather than a linear pathway. In neutrophils, GSDMD pore formation permits the membrane permeabilization that precedes mtDNA externalization, so that oxidized mtDNA released through this route can promote further GSDMD oligomerization in neighboring cells (176). This axis is engaged according to the oxidation state of mtDNA rather than its abundance, consistent with the broader observation that immunostimulatory potency in SLE is governed principally by chemical modification.

In summary, the cytoplasmic ectopic redistribution of mtDNA in SLE is not a passive consequence of mitochondrial injury but an active and self-amplifying pathological process that drives disease initiation, perpetuation, and chronicity. From ultrastructural mitochondrial remodeling that destabilizes nucleoid anchoring, to mPTP-mediated trans-membrane release, to mitophagy failure enabling accumulation of oxidized immunogenic mtDNA, to mtDNA-driven innate immune activation with multilayered reciprocal feedback, each mechanistic module, anchored on mtDNA as the central node, is tightly interconnected within a pathophysiological network that is both precisely regulated and inherently prone to self-reinforcing escalation.

#### 3.2.2 Extracellular release of mitochondrial DNA

The translocation of mtDNA into the extracellular space marks a critical inflection point in SLE pathophysiology, representing the transition from cell-autonomous inflammatory responses to systemic immune dysregulation that engages diverse tissue compartments and cell lineages. Extracellular mtDNA exists in multiple molecular forms, including free double-stranded or single-stranded DNA, nucleoprotein complexes, membrane-enclosed vesicles such as exosomes and microparticles, lipid-associated complexes, and fragmented free DNA (67, 177, 178), and compared with nuclear DNA, circulating mtDNA displays markedly prolonged circulatory half-life and enhanced serum stability (179, 180). Under select pathophysiological stress conditions, mtDNA can be released either as free DNA or as TFAM-associated complexes from diverse immune and non-immune cell populations, stimulating dendritic cells and macrophages and inducing dose-dependent increases in pro-inflammatory cytokine production (37, 181, 182).

Neutrophil extracellular trap (NET) formation represents a quantitatively major pathway for extracellular mtDNA release in SLE. Lood et al. demonstrated that plasma concentrations of MPO-DNA complexes, a canonical NET-associated biomarker, are markedly elevated in SLE, with a substantial proportion of the nucleic acid component originating from the mitochondrial genome (183). During NETosis, activated neutrophils undergo a distinctive lytic cell death program in which nuclear chromatin and mtDNA are coordinately extruded and assembled into three-dimensional reticular scaffolds decorated with antimicrobial peptides, post-translationally modified histones, and myeloperoxidase (184, 185). Notably, mtDNA embedded within NET structures forms thermodynamically stable nucleoprotein complexes with histone H4, and these aggregates display substantially enhanced pro-inflammatory activity with the capacity to activate diverse immune cell populations across multiple lineages (186). Secretion of mtDNA-TFAM complexes into the extracellular space also occurs as a component of normal neutrophil biology (183, 187); in SLE, however, reduced DNase I activity and the presence of anti-DNase I neutralizing autoantibodies impair NET degradation, leading to pathological NET accumulation (188). Lood et al. further demonstrated that ribonucleoprotein immune complexes (RNP IC) trigger mitochondrial membrane potential dissipation and surface translocation in neutrophils, driving ROS-dominated NETosis with extracellular release of oxidized mtDNA, a mechanism that induces lupus-like phenotypes with prominent type I interferon signatures in murine models (183). Low-density granulocytes (LDGs) circulating in SLE peripheral blood exhibit an exaggerated propensity for NET elaboration, further amplifying this process (189). Sustained NET-associated mtDNA release is associated with heightened type I interferon production and interferon-stimulated gene (ISG) transcriptional programs, which may in turn promote additional mtDNA release. The existence of a fully self-perpetuating loop remains inferred rather than directly demonstrated.

Programmed cell death and necrosis contribute substantially to extracellular mtDNA release through distinct but complementary mechanisms. The defective apoptotic cell clearance that characterizes SLE leads to pathological accumulation of late-stage apoptotic and necrotic cellular debris (6, 190), whose cytoplasmic contents include both native and oxidatively modified mtDNA (169, 191). Beyond passive release through cell death, discrete metabolically active cell populations can also secrete mtDNA through deliberate extracellular vesicular trafficking pathways including exosomal and microvesicular biogenesis, an observation consistent with elevated mtDNA content in serum-derived exosomes from SLE patients relative to healthy controls (99, 192, 193).

**Figure 2:**
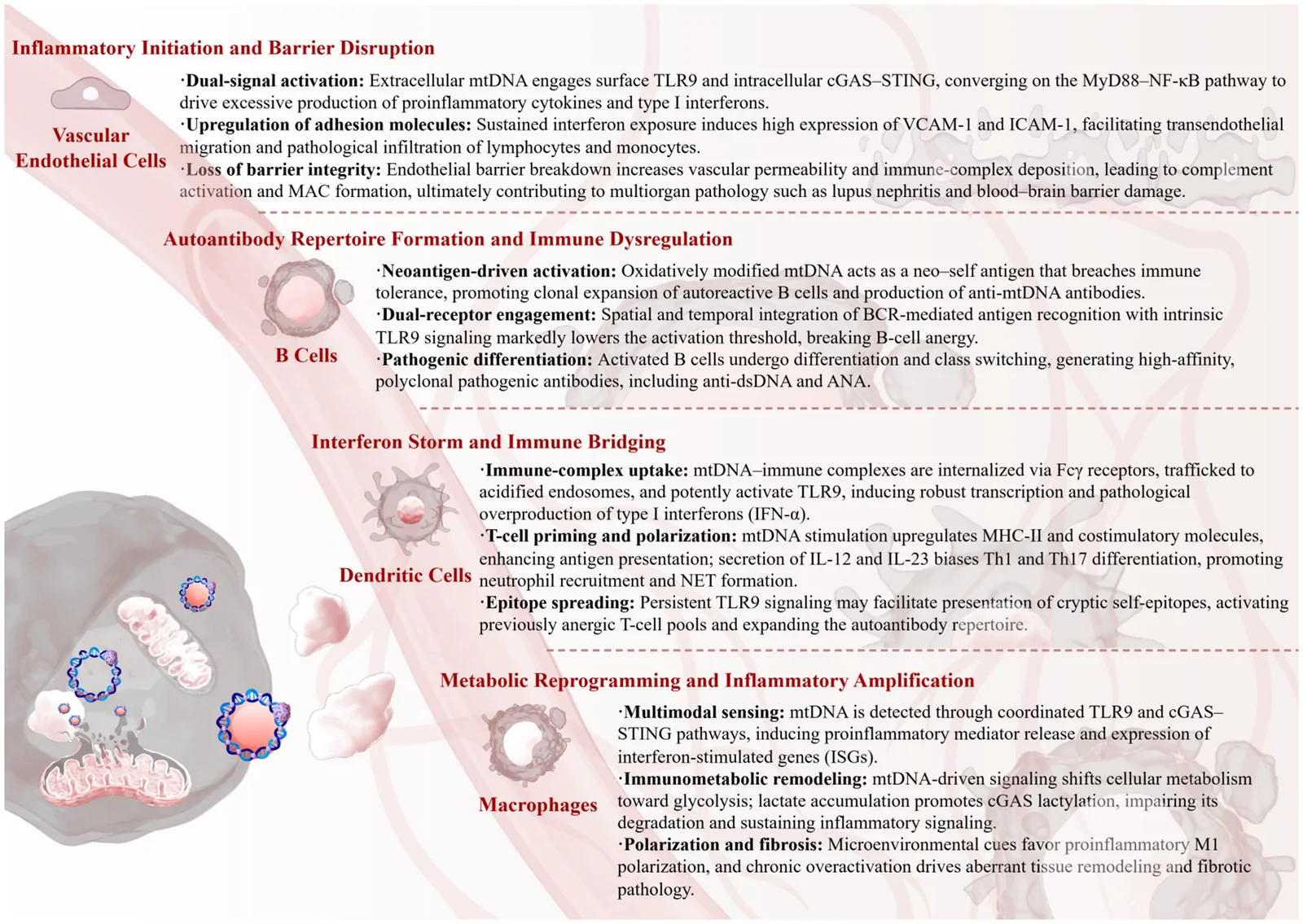
mtDNA-driven multi-lineage immune activation and assembly of the SLE inflammatory circuit. Under the pathophysiological conditions of SLE, extracellular mtDNA functions as a potent DAMP that enters target cells via endocytic internalization or receptor-mediated uptake, initiating coordinated inflammatory cascades across multiple immune compartments. In vascular endothelial cells, mtDNA engages both TLR9 and the cGAS-STING axis, activating NF-κB and type I interferon transcriptional programs that upregulate adhesion molecules and progressively disrupt endothelial barrier integrity, thereby facilitating pathological leukocyte extravasation into the vessel wall. In B lymphocytes, oxidatively modified mtDNA acts as a cryptic neoantigen that exploits a dual-receptor mechanism, in which simultaneous BCR and TLR9 co-engagement converges to breach immunological tolerance checkpoints and drive clonal expansion toward a pathogenic autoantibody repertoire. Plasmacytoid dendritic cells (pDCs), upon Fcγ receptor-mediated acquisition of mtDNA-containing immune complexes, mount robust type I interferon responses that sustain the interferon signature characteristic of SLE, while concurrently promoting adaptive immune escalation through epitope spreading and preferential Th1/Th17 lineage commitment. Macrophages undergo significant metabolic reprogramming upon mtDNA sensing, characterized by a Warburg-like shift toward glycolysis and cGAS lactylation-dependent signal amplification, which together reinforce sustained pro-inflammatory M1 polarization and accelerate fibrogenic tissue remodeling. The coordinated activation of these diverse cellular populations assembles a self-amplifying inflammatory circuit that collectively drives the multi-organ involvement and progressive tissue injury that define SLE.

As a conserved DAMP, extracellular mtDNA is internalized by target cells through clathrin-dependent endocytosis, phagocytic engulfment, and receptor-mediated endocytic pathways (176, 194, 195); direct trans-membrane diffusion across the lipid bilayer is negligible given DNA’s intrinsic polyanionic character (196, 197). Once internalized and immunologically sensed, mtDNA initiates cascading inflammatory responses that engage multiple immune cell lineages in building self-reinforcing inflammatory networks (**Figure 2**).

Vascular endothelial cells, positioned at the critical interface between the circulation and tissue interstitium, are highly sensitive to circulating mtDNA signals (170, 198, 199). In SLE, extracellular mtDNA is recognized by endothelial TLR9 to activate the MyD88-NF-κB transcriptional cascade (200), while the cytosolic cGAS-STING axis operates in parallel to drive type I interferon production (201). Sustained interferon exposure upregulates the endothelial adhesion molecule repertoire, including VCAM-1, ICAM-1, and E-selectin, promoting aberrant transendothelial migration of autoreactive lymphocytes and inflammatory monocytes into the vascular wall (202–204). Progressive disruption of endothelial barrier integrity may contribute to lupus vasculitis but also creates pathological conditions for immune complex deposition at vascular basement membranes, triggering complement activation and membrane attack complex (MAC) assembly, and ultimately driving organ-specific injury including glomerular basement membrane lesions in LN, dermal-epidermal junction disruption in cutaneous lupus erythematosus (CLE), and blood-brain barrier compromise in neuropsychiatric SLE (NPSLE) (205).

B cell dysregulation is a defining immunological hallmark of SLE, and mtDNA plays mechanistically distinct but convergent roles in driving pathogenic autoantibody production. Extracellular mtDNA, particularly oxidatively modified forms carrying structurally altered neo-epitopes capable of breaching central and peripheral immune tolerance checkpoints, stimulates clonal expansion and differentiation of autoreactive B cells, sustaining pathological autoantibody biosynthesis (120, 148). Anti-mtDNA antibodies detectable in SLE sera predominantly target the D-loop region and conserved coding sequences, correlating with interferon gene signatures and standardized disease activity indices (120–122). Furthermore, B cells in SLE, particularly marginal zone (MZB) and B-1 cell subsets, express abnormally elevated levels of TLR9 and exhibit pathological hyper-responsiveness to mtDNA stimulation (123, 206). When mtDNA co-localizes with nucleosomal particles, histone octamers, or ribonucleoprotein complexes, these multi-molecular assemblies are efficiently endocytosed by autoreactive B cells through antigen-specific BCR recognition, whereupon mtDNA provides potent TLR9-mediated co-stimulatory signals within the endosomal compartment, substantially lowering the threshold for productive BCR activation (207, 208). This dual-receptor mechanism, integrating BCR-transduced antigen-specific signals with TLR9-mediated innate immune signals, represents the decisive molecular event through which autoreactive B cells overcome clonal anergy and acquire full activation, subsequently producing a spectrum of pathogenic autoantibodies including anti-dsDNA, ANA, anti-Sm, and anti-SSA/SSB antibodies, as well as high-affinity, class-switched IgG species with direct pathogenic consequences.

Dendritic cells sense extracellular mtDNA through both TLR9 and cGAS-STING pathways (37). Upon endocytic uptake of mtDNA, pDCs undergo TLR9-driven maturation programs characterized by marked upregulation of co-stimulatory molecules (CD80, CD86, CD40) and MHC-II, enhancing their capacity to prime antigen-specific T cell responses (209). The resulting cytokine secretion profile, dominated by IL-12 and IL-23, promotes differentiation of naïve T cells toward Th1 and Th17 effector lineages (210, 211); IL-17 produced by Th17 cells in turn drives neutrophil recruitment and NET elaboration, with the additional mtDNA liberated by neutrophils engaging pDC TLR9 to sustain type I interferon production and close another amplification loop (182). Oxidatively modified mtDNA is substantially more immunostimulatory than its unmodified counterpart (144, 169), and activated lupus neutrophils, or normal neutrophils stimulated by IFN-α/RNP, inhibit cAMP-PKA-mediated TFAM phosphorylation, impairing mtDNA nucleoid packaging such that oxidized mtDNA is expelled in interferon-potentiating complexes that drive elevated IFN-α production in pDCs (212). Beyond these immediate effects, sustained mtDNA-TLR9 signaling may enable DCs to present previously cryptic self-epitopes, activating anergic or immunologically naïve autoreactive T cell clones and driving epitope spreading, a key mechanism underlying the progressive broadening of autoantibody specificities over the course of SLE (213–215).

pDCs, as specialized high-capacity type I interferon-producing cells, are particularly sensitive to circulating mtDNA-immune complex conjugates (136, 216). When anti-DNA or ANA antibodies form immune complexes with extracellular mtDNA, these assemblies are efficiently internalized by pDCs via Fcγ receptor-mediated endocytosis, a trafficking route that preferentially delivers cargo to endosomal-lysosomal compartments rather than autophagic degradation pathways, causing co-localization of mtDNA with endosomal TLR9 and triggering explosive interferon transcriptional activation (214). Immune complex-associated mtDNA is internalized more efficiently than free mtDNA, with antibody complexation concentrating mtDNA within endosomal compartments and amplifying focal TLR9 engagement and downstream signaling intensity (217).

Macrophages express a broad repertoire of PRRs capable of mtDNA recognition, constituting a multi-layered immune surveillance network in which synergistic activation of the TLR9-MyD88 and cGAS-STING axes drives robust pro-inflammatory mediator release and ISG expression (218). Under mtDNA stimulation, macrophages undergo metabolic reprogramming toward enhanced glycolytic flux, and the resulting lactate accumulation mediates cGAS lactylation that antagonizes its ubiquitin-mediated degradation and amplifies cGAS-STING-driven interferon and cytokine production (156). Macrophage phagocytosis can additionally internalize intact mitochondria released from necroptotic cells, incorporating their mtDNA cargo into intracellular compartments (219); mtDNA-derived signals preferentially polarize macrophages toward pro-inflammatory M1 states or programmed cell death outcomes (220, 221), and chronic mtDNA exposure sustains macrophage hyperactivation and progressive fibrotic tissue remodeling (222).

Taken together, the sequential stages of cytoplasmic mtDNA translocation and extracellular release are tightly coupled across spatiotemporal dimensions, synergistically driving the diverse pathological processes of SLE. Extracellular mtDNA coordinates a highly integrated, multi-lineage inflammatory network spanning endothelial activation and vascular remodeling, B cell autoreactivity and autoantibody production, DC antigen presentation and T cell instructional dysregulation, and macrophage-mediated inflammatory amplification. By engaging both endosomal pattern recognition receptors, primarily TLR9 and STING, and extracellular receptor-mediated pathways including Fc receptor-directed uptake in macrophages and DCs, mtDNA dynamically couples these diverse cellular populations through cytokine networks and intercellular contact-dependent signaling, assembling an evolving inflammatory regulatory architecture that substantially deepens and perpetuates the immune dysregulation characteristic of SLE (**Table 2**).

**Table 2:** Mechanisms of mtDNA cytoplasmic and extracellular release and the orchestrated immune activation network in SLE pathogenesis.

Release stage	Release pathway	Key molecular mechanisms	mtDNA form	Immune receptors	Downstream signaling	Major effector cells	Pathological outcomes
Cytoplasmic Translocation	Mitochondrial Ultrastructural Remodeling	Matrix swelling→inner membrane anchorage site dissociation; cristae disintegration→cardiolipin microdomain dissolution	Free nucleoids	cGAS	cGAS-STING-TBK1-IRF3/NF-κB	T cells, monocytes	Type I IFN storm
	Pathological mPTP Opening	VDAC1/3 oligomerization; CypD oxidative modification; matrix Ca^2+^ overload	Oxidized mtDNA fragments	cGAS, TLR9	MyD88-NF-κB; STING pathway	Multiple immune cells	Pro-inflammatory cytokine cascade
	Autophagy dysfunction	IFN-ISG15ylation inhibition; lysosomal alkalinization; autophagosome-lysosome fusion impairment	Accumulated Ox-mtDNA	TLR9, NLRP3	Inflammasome activation	Monocytes, macrophages	IL-1β/IL-18 maturation and secretion
	Oxidative Stress	TFAM oxidation and Lonp1 degradation→nucleoid depolymerization	Exposed unmethylated CpG-mtDNA	cGAS, TLR9	Dual signal activation	PBMCs	Self-amplifying inflammatory circuit
Extracellular Release	NETs	NETosis with mtDNA extrusion	mtDNA–TFAM complexes in NETs	TLR9	pDC high IFN-α secretion	Neutrophils, pDCs	Sustained IFN activation; low-density granulocyte expansion
	Programmed cell death	Defective apoptotic clearance	Native & Ox-mtDNA	TLR9, cGAS, AIM2	Multiple DAMP pathways	Macrophages, DCs	Chronic inflammation
	Extracellular vesicles	Exosome-packaged mtDNA	Membrane-encapsulated mtDNA	TLR9 after endocytosis	Endosomal TLR9 activation	Metabolic cells	Intercellular inflammatory signal transmission

### 3.3 Organ-specific pathological sequelae

mtDNA-mediated injury mechanisms constitute an integral pathophysiological dimension of the organ-specific damage that defines the clinical course of SLE.

#### 3.3.1 Lupus nephritis: renal parenchymal injury

As a metabolically demanding organ with exceptionally high bioenergetic requirements, the kidney is particularly vulnerable to mtDNA-mediated injury in the context of LN (223, 224). Podocytes and renal tubular epithelial cells in LN patients exhibit quantitative mitochondrial depletion, with the majority of residual mitochondria displaying characteristic morphological abnormalities including matrix swelling and cristae fragmentation, changes that directly impair glomerular filtration capacity and tubular solute transport function (135, 223). Aberrantly released mtDNA engages TLR9 in podocytes, compromising mitochondrial bioenergetic function, inducing foot process effacement and glomerular basement membrane thickening, and thereby abolishing the structural basis for proteinuria prevention (201, 225). In experimental models, mtDNA release activates the cGAS-STING pathway, sustaining type I interferon signaling that amplifies inflammatory transcriptional programs, drives chemokine-mediated leukocyte recruitment, and is associated with progressive interstitial fibrosis (98, 201). mtDNA also triggers NLRP3 inflammasome-dependent pyroptosis in renal parenchymal cells, substantially reducing cellular viability and contributing to ongoing parenchymal loss (226, 227).

At the clinical level, circulating cfDNA concentrations correlate robustly with nephropathic involvement in SLE (98, 119). Comprehensive analysis of four cfDNA molecular parameters, encompassing fragmentation index, intracellular-to-extracellular mtDNA copy number ratio, absolute cfDNA concentration, and the presence of 54–149 bp and 209–297 bp oligonucleotide fragments, reveals that patients with physiologically normal cfDNA profiles demonstrate superior eGFR preservation and are less likely to require renal biopsy or develop progressive LN; conversely, those with aberrant cfDNA signatures show substantially more severe eGFR deterioration, a higher prevalence of progressive LN phenotypes, and greater rates of renal transplantation (117). Histopathological examination of LN renal biopsies demonstrates increased NET deposition with prominent intra-NET mtDNA accumulation (121, 228). These accumulated NET structures exacerbate glomerular mesangial proliferation and basement membrane thickening, become entrapped within peritubular capillaries, and drive tubulointerstitial inflammatory cascades (176). As parenchymal injury progresses, further deterioration of mitochondrial homeostasis is likely to result in additional mtDNA release, though the resulting amplification circuit has been inferred rather than directly measured in patient tissue. Oxidized mtDNA can also induce GSDMD oligomerization, a prerequisite molecular step for neutrophilic mtDNA extrusion (176).

Clinical evidence further demonstrates that serum anti-mtDNA antibody titers correlate with LN severity more strongly than anti-dsDNA titers, and that mtDNA/anti-mtDNA immune complexes induce pDC-IFN-α axis dysregulation through TLR-mediated signaling (121). mtDNA deletion mutation frequency correlates positively with the degree of renal functional impairment, and patients carrying elevated mtDNA D-loop D310 variants show an increased propensity for nephropathic manifestations (112). Collectively, these observations support the premise that indices of mtDNA mutation burden and oxidative damage hold significant potential as prognostic biomarkers for renal functional deterioration in SLE, a point elaborated further in the integrated biomarker framework presented in Section 3.1.1.

#### 3.3.2 Neuropsychiatric SLE: neuroinflammatory dysregulation

The neurological manifestations of NPSLE span a heterogeneous clinical spectrum, ranging from cognitive impairment and mood disorders to epileptic seizures and frank psychosis. Blood-brain barrier disruption provides pathophysiological conduits through which circulating mtDNA and inflammatory mediators gain access to cerebral microvascular endothelial cells and neural parenchyma (229). The hippocampus is particularly susceptible to mtDNA-mediated neuroinflammatory injury, a vulnerability that may partly explain the high prevalence of memory deficits and cognitive dysfunction observed in SLE patients (230, 231). Among cerebral cell populations, microglia exhibit heightened sensitivity to mtDNA, responding to TLR9 and cGAS-STING engagement with pro-inflammatory polarization, robust production of TNF-α, IL-6, and ROS, and consequent direct neuronal cytotoxicity (232, 233). The intrinsically high bioenergetic demands of neurons render them susceptible to mitochondrial structural compromise and local mtDNA release under conditions of ischemia, hypoxia, or excitotoxic glutamate stimulation, locally amplifying neuroinflammatory cascades (234). Choroid plexus epithelial cells respond to mtDNA stimulation by altering cerebrospinal fluid composition, thereby disrupting the homeostatic microenvironment of the central nervous system (235). Consistent with these mechanisms, elevated cerebrospinal fluid mtDNA concentrations correlate with standardized neurocognitive assessment scores and with disease activity indices and symptom burden in NPSLE patients (236).

#### 3.3.3 Cutaneous lupus erythematosus: UV-induced inflammatory cascades

In the context of SLE photosensitivity, ultraviolet radiation exposure directly augments epidermal cell structural damage, promoting mtDNA strand breaks, release, and oxidative modification (237, 238). Following keratinocyte-derived mtDNA extrusion, skin-resident pDCs are activated through TLR9 and cGAS-STING engagement, establishing a high-interferon dermal microenvironment that contributes to epidermal cytotoxicity and promotes immune effector cell infiltration into cutaneous tissue, generating the histopathological changes characteristic of interface dermatitis (201, 239–241). NET formation is additionally observed in cutaneous lesional tissue, with neutrophils releasing mtDNA into the extracellular space through chromatin externalization, thereby sustaining the type I interferon transcriptional signature that typifies SLE (99, 242, 243).

#### 3.3.4 Cardiovascular involvement: endothelial and vascular injury

In the cardiovascular system, circulating mtDNA acts on vascular endothelial cells to increase endothelial permeability and induces pro-inflammatory phenotypic transformation. Macrophages derived from monocytic precursors under sustained mtDNA stimulation conditions can undergo foam cell transformation upon phagocytosis of oxidized low-density lipoprotein particles, accelerating atheromatous plaque formation and representing a key pathophysiological mechanism underlying the heightened cardiovascular risk characteristic of SLE (218). Circulating mtDNA levels are elevated in SLE patient cohorts, particularly in those with traditional cardiovascular risk factors or subclinical atherosclerosis, a phenomenon partly attributable to the clonal expansion of SLE-associated low-density granulocytes (LDGs) (189). Defective mitochondrial clearance during erythropoietic maturation in SLE patients leads to the accumulation of mitochondria-retaining erythrocytes that are phagocytosed by macrophages, triggering intracellular mtDNA-cGAS pathway activation and type I interferon induction (57, 244). In murine models, Fcγ receptor IIA-mediated platelet activation drives intracellular mtDNA liberation and exacerbates lupus phenotypic manifestations (195), and activated platelet exocytosis represents an additional significant source of circulating mtDNA in SLE patients (99).

#### 3.3.5 Pulmonary involvement: interstitial pneumopathic manifestations

Pulmonary involvement in SLE encompasses interstitial lung disease, pleuritis, and related pneumopathic manifestations. Although the overall prevalence of pulmonary complications is relatively modest, alveolar epithelial cells under pathophysiological stress conditions release mtDNA that activates alveolar macrophages toward pro-fibrotic mediator production, initiating progressive pulmonary fibrosis (245, 246). In models of acute lung injury, mtDNA promotes pulmonary microvascular inflammatory activation and increases vascular permeability, changes that may culminate in interstitial edema. Whether this mechanism operates in lupus-associated interstitial lung disease has not been established (246, 247).

#### 3.3.6 Articular involvement: synovial inflammation and joint pathology

Joint involvement is the most frequently encountered clinical manifestation of SLE, affecting the majority of patients over the disease course and ranging from intermittent arthralgia to non-erosive Jaccoud arthropathy. Despite this clinical prominence, the mitochondrial contribution to lupus synovitis has received considerably less mechanistic attention than renal or cutaneous disease, and the available evidence derives substantially from studies of other inflammatory arthropathies.

Synovial fibroblasts appear to be a cell population in which mitochondrial integrity directly governs inflammatory output. In normal human synoviocytes, experimental disruption of mitochondrial function raises both cytosolic and mitochondrial ROS, and this shift is accompanied by NF-κB activation together with increased expression of COX-2, prostaglandin E2, and IL-8, indicating that mitochondrial dysfunction alone is sufficient to establish a low-grade inflammatory phenotype in cells that were not otherwise stimulated (248). The same cells show amplified responses when subsequently exposed to pro-inflammatory cytokines, suggesting that mitochondrial impairment lowers the activation threshold of the synovial compartment rather than simply adding an independent inflammatory signal (249). Given that ROS overproduction and mitochondrial fragmentation are documented features of SLE immune cells, it is plausible that a comparable priming effect operates within the lupus synovium, although this has not been directly demonstrated.

Evidence implicating mtDNA itself is more specific in rheumatoid arthritis. Extracellular mtDNA together with oxidatively damaged DNA has been detected in the synovial fluid of patients with rheumatoid arthritis but not in that of healthy individuals (250, 251), and intra-articular administration of oxidatively modified mtDNA is sufficient to elicit arthritis in murine models, with concomitant induction of TNF-α from synovial cells (252). Somatic mtDNA mutations have additionally been identified in rheumatoid synoviocytes, consistent with cumulative oxidative injury within a chronically hypoxic and metabolically stressed joint microenvironment (253). Because the innate sensing machinery engaged by these species, principally TLR9 and cGAS-STING, is the same machinery shown to be hyperresponsive to mtDNA in SLE, the synovium represents a biologically coherent site at which the mtDNA-driven circuits described in preceding sections could operate.

This coherence should nonetheless be stated as a hypothesis rather than an established mechanism. To our knowledge, no study has quantified synovial fluid mtDNA in patients with lupus arthritis, characterized mtDNA copy number or oxidative burden in lupus synoviocytes, or tested whether articular manifestations track with the circulating mtDNA parameters. The non-erosive character of lupus arthritis also distinguishes it pathologically from rheumatoid disease, so mechanisms established in the latter cannot be transposed without qualification. Direct interrogation of the lupus synovium therefore represents one of the more tractable and clinically relevant gaps in this field, and would determine whether articular involvement belongs within the mtDNA-driven framework proposed here or reflects a mechanistically distinct process.

**Figure 3:**
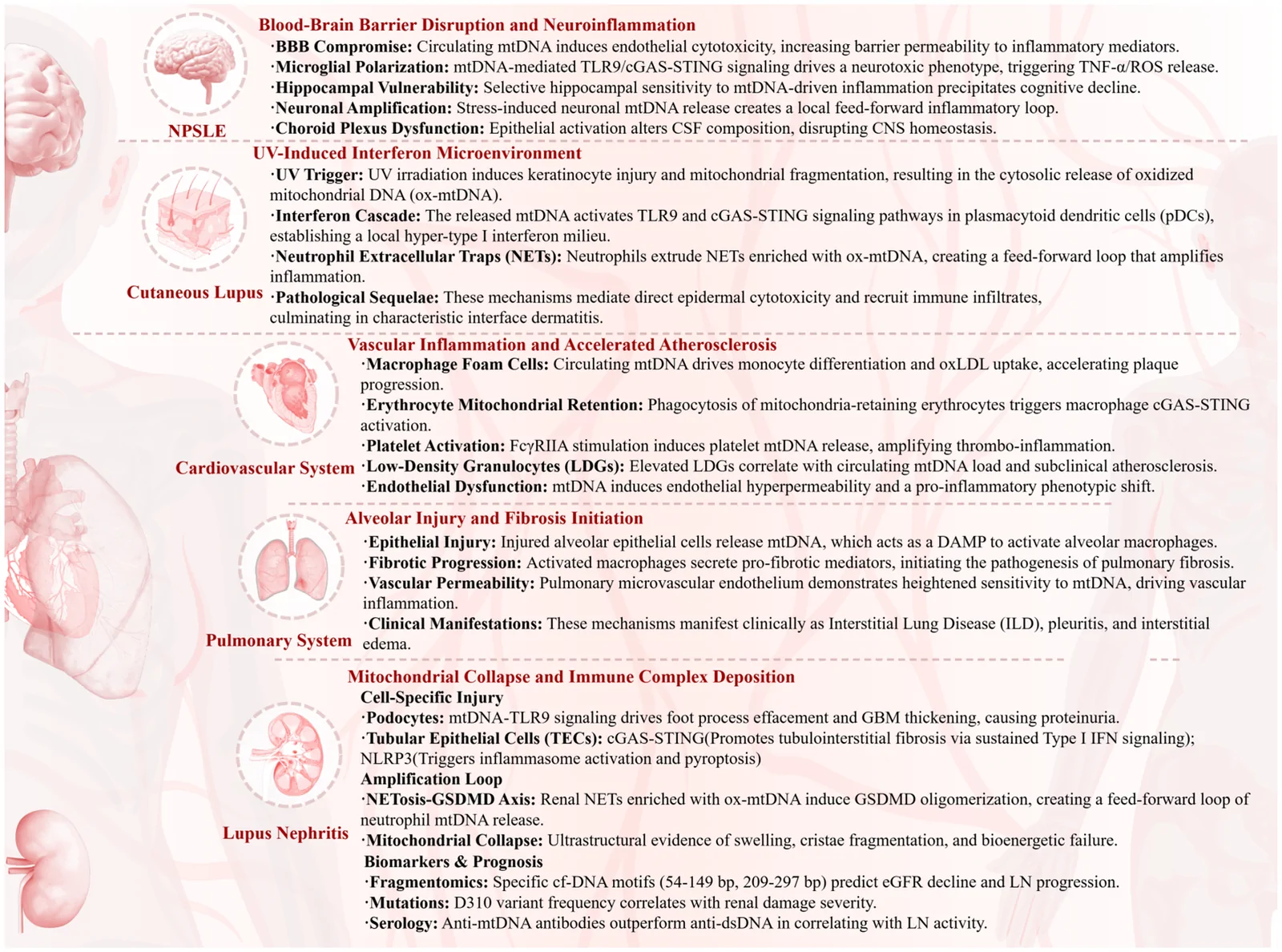
mtDNA-mediated mechanisms of multi-organ injury in SLE. mtDNA, acting as a central DAMP in SLE, drives organ-specific dysfunction through a network of interconnected inflammatory signaling cascades. Within the renal compartment, aberrant mtDNA release and NET deposition in podocytes and tubular epithelial cells activate TLR9 and the cGAS-STING axis, resulting in podocyte foot process effacement, pronounced inflammatory infiltration, and progressive interstitial fibrosis, with circulating cell-free DNA molecular signatures correlating robustly with the degree of renal functional decline. Neurological manifestations arise from blood-brain barrier disruption that permits circulating mtDNA to access the cerebral parenchyma, where it activates microglia and initiates neuroinflammatory cascades that impair neurocognitive function. Cutaneous pathology is driven by ultraviolet-induced mtDNA release from keratinocytes, which primes skin-resident pDCs to establish a high-interferon tissue microenvironment and precipitates the interface dermatitis characteristic of cutaneous lupus. Cardiovascular involvement is characterized by mtDNA-driven macrophage foam cell transformation and endothelial dysfunction, collectively accelerating atherogenesis and increasing cardiovascular risk. Pulmonary pathology manifests as pro-fibrotic alveolar remodeling and microvascular endothelial injury leading to interstitial edema. Critically, these organ-specific injuries do not occur as isolated events but constitute an integrated pathological network, in which circulating mtDNA perpetuates a systemic inflammatory amplification loop that synergistically drives and sustains the multisystem pathological trajectory of SLE.

It is important to recognize that although the organ-specific mechanisms described above are discussed separately, they do not operate in isolation. Rather, they unfold within a framework of systemic inflammatory interdependencies and inter-organ crosstalk, in which mtDNA released from one organ system exerts pathophysiological effects on distant organs through hematogenous circulation, establishing cascading multi-organ injury dynamics. As an illustrative example, mtDNA released as a consequence of renal injury can exacerbate vascular endothelial damage, while vascular inflammatory pathology in turn impairs renal hemodynamics and functional capacity. These bidirectional interactions contribute to the clinical complexity and temporal variability that characterize SLE disease trajectories. Individual genetic variation in mtDNA immunological responsiveness further influences patient-specific susceptibility to mtDNA-driven inflammation and the distribution of organ involvement across patients (**Figure 3**). The depth of evidence differs markedly across these organ systems. Renal involvement is supported by patient biopsy material, biomarker correlations, and mechanistic work in human cells; the neuropsychiatric and pulmonary accounts are assembled largely from non-lupus contexts, with the mtDNA contribution inferred from shared sensing pathways rather than demonstrated in lupus tissue. This asymmetry reflects the accessibility of tissue for study more than the true distribution of injury.

## 4 Current status and prospects of mtDNA-targeted therapeutic strategies

### 4.1 Overview of mtDNA-directed therapeutic modalities

Therapeutic strategies targeting mtDNA can be deployed at multiple levels of the mitochondrial damage cascade. Antioxidant-based interventions represent the most direct and extensively investigated approach. Conventional antioxidants, including vitamin E, vitamin C, and glutathione, possess inherent free radical scavenging activity but demonstrate limited efficacy in protecting mtDNA, primarily because they fail to accumulate within mitochondria at therapeutically meaningful concentrations (254, 255). To overcome this pharmacokinetic limitation, investigators have developed mitochondria-targeted antioxidant platforms, of which coenzyme Q10 is the most well-characterized (256, 257). As an integral component of the electron transport chain, coenzyme Q10 exerts direct antioxidant activity within the mitochondrial matrix, scavenging ROS and reducing oxidative insults to mtDNA. MitoQ, another prominent mitochondria-targeted antioxidant, achieves selective mitochondrial enrichment through covalent conjugation of a coenzyme Q10 moiety with a lipophilic triphenylphosphonium cation, exploiting the electrochemical gradient across the inner mitochondrial membrane to concentrate the compound within the matrix and thereby provide enhanced mtDNA protection (258, 259). Related compounds, including MitoTEMPO and SkQ1, are founded on the same principle of conjugating antioxidant moieties to lipophilic cationic scaffolds (260).

A second major therapeutic category involves promotion of mitochondrial biogenesis to improve both mtDNA quality and quantity. Physical exercise (261), resveratrol (262), and metformin (263) each activate the PGC-1α signaling cascade through distinct upstream mechanisms. NAD+ is a central coenzyme in mitochondrial energy metabolism and a key signaling molecule regulating mitochondrial biogenesis, and tissue NAD+ levels decline progressively with aging and in pathological states (264). Supplementation with NAD+ precursors, most notably nicotinamide riboside (NR) and nicotinamide mononucleotide (NMN), raises intracellular NAD+ pools, activates the deacetylase SIRT1, and stimulates mitochondrial biogenesis while enhancing mtDNA quality control mechanisms (265, 266); these compounds additionally activate PARP family proteins, which coordinate DNA damage repair processes including mtDNA repair pathways (267, 268).

Direct pharmacological modulation of mtDNA repair machinery constitutes a third therapeutically relevant approach. Although the mitochondrial DNA repair apparatus is less elaborate than its nuclear counterpart, the BER pathway remains functionally operative. OGG1, the primary mitochondrial DNA glycosylase, recognizes and excises oxidatively damaged guanine bases to initiate BER, and strategies to enhance OGG1 activity or expression levels can augment overall mtDNA repair capacity (269). The mitochondria-specific helicase Twinkle (270) and mitochondrial DNA polymerase γ (271) are additional targets of interest, given their central roles in mtDNA replication and genomic maintenance.

More experimental therapeutic approaches include mitochondrial transplantation and gene therapy. Mitochondrial transplantation involves the delivery of functional exogenous mitochondria to compromised cells or tissues via direct injection, co-culture systems, or nanocarrier-mediated delivery, with the aim of replacing or supplementing dysfunctional organelles (272, 273). Preclinical studies have demonstrated proof-of-concept in several disease models, including idiopathic inflammatory myopathy (274), myocardial ischemia (275), spinal cord injury (276), and PD (277), though the approach faces substantial practical challenges encompassing mitochondrial procurement, purification, preservation, and post-transplantation viability, integration, and functional reconstitution. With respect to gene therapy, the development of platforms for mtDNA-specific genetic modification has proceeded more slowly than for nuclear genome editing, largely because the mitochondrial double-membrane architecture presents formidable barriers to the intramitochondrial delivery of macromolecular editing tools such as CRISPR-Cas9 (278). Investigators have nonetheless explored several strategies, including the use of mitochondria-targeting sequences to direct editing machinery into the organelle and the exploitation of endogenous mitochondrial protein import pathways (279–281). An alternative approach involves indirectly influencing mitochondrial function and mtDNA stability through nuclear genome editing, targeting genes that regulate mitochondrial biogenesis, dynamics, or antioxidant defense (282, 283). Antisense oligonucleotide technology has also been applied to modulate mitochondrial gene expression, although intramitochondrial delivery efficiency remains a significant limiting factor (284, 285).

### 4.2 Clinical evidence for mtDNA-targeted interventions in SLE

Among the range of mtDNA-targeted therapeutic strategies, those with rigorous clinical trial validation in SLE remain limited, concentrated primarily in the domains of antioxidant therapy and metabolic modulation. N-acetylcysteine (NAC) currently holds the strongest clinical evidence base among mitochondria-related therapeutic agents investigated in SLE (286). As a glutathione biosynthetic precursor, NAC raises intracellular glutathione levels and exerts broad antioxidant activity (287, 288). In a clinical trial conducted by Lai et al. at the University of Virginia, daily NAC supplementation in SLE patients reduced SLEDAI scores and markedly elevated glutathione concentrations in patient-derived T lymphocytes, accompanied by correction of oxidative stress biomarkers and mitochondrial hyperpolarization (289). Subsequent investigations demonstrated that NAC also improves flow-mediated vascular dilation and reduces fatigue scores, suggesting that in addition to its protective effects on mtDNA, NAC confers benefits on vascular endothelial function with potential implications for cardiovascular risk reduction in SLE (290). These studies are nonetheless limited by small sample sizes, insufficient long-term follow-up, incomplete inclusion of patients with severe organ involvement, and a higher-than-anticipated incidence of gastrointestinal adverse effects.

Clinical evidence supporting coenzyme Q10 in SLE remains limited, with its proposed role in mtDNA homeostasis preservation largely theoretical. In preclinical studies using the MRL/lpr murine model, coenzyme Q10 ameliorated multiple pathological features through improvements in mitochondrial homeostasis, attenuation of pro-inflammatory cytokine production, and suppression of NET formation, with particularly pronounced effects on renal and vascular endothelial phenotypes (291). Clinical translation has not, however, advanced convincingly, and the intrinsically poor bioavailability of coenzyme Q10 remains a significant obstacle that must be addressed before meaningful clinical implementation is feasible. MitoQ, as a second-generation mitochondria-targeted antioxidant, has demonstrated favorable safety profiles in multiple clinical trials conducted in neurodegenerative (292, 293) and cardiovascular disease settings (294, 295); however, formal clinical trial data in SLE patients are absent, and foundational preclinical efficacy studies in SLE animal models have not been reported. Substantial additional evidence and safety data will be required before MitoQ can be considered for clinical translation in SLE.

The investigation of metformin in SLE illustrates a recurring pattern in this field: a substantial body of preclinical and retrospective clinical data that has not yet been matched by prospective randomized trial evidence. In lupus murine models, metformin has been shown to reduce renal pathology, proteinuria, immune complex deposition, and mortality, through mechanisms including AMPK activation, mTOR suppression, improved mitochondrial function, and immune metabolic reprogramming (296–299). Clinical findings have been less consistent. A proof-of-concept trial in 113 patients reported that metformin added to corticosteroids and conventional immunosuppressants attenuated renal NET formation and pDC-IFN-α pathway activation, with variable reductions in flare frequency and cumulative prednisone exposure in mild-to-moderate disease (121). Analyses by Ye and colleagues identified patients with lower disease activity, shorter disease duration, and hydroxychloroquine maintenance as most likely to benefit, with those in serological remission responding most favorably (300), and subsequent work from the same group found earlier and more sustained attainment of treat-to-target endpoints relative to controls (301). A multicenter, randomized, double-blind, placebo-controlled trial in 140 non-diabetic adult patients, however, found no significant effect on the primary endpoint of SELENA-SLEDAI flare index (302).

These discrepant results may reflect an underappreciated feature of the drug itself. Metformin’s effects on mitochondria are bidirectional: moderate complex I inhibition activates AMPK and may promote mitochondrial biogenesis over extended treatment, but acutely reduces mitochondrial ATP production. In patients whose mitochondrial function is already severely compromised, this inhibitory action could plausibly be counterproductive, which would be consistent with benefit concentrating in the lower-activity subgroups identified above. Resolving whether metformin is broadly ineffective or effective only in defined subpopulations will require prospective trials with prespecified stratification rather than further *post hoc* analysis.

NAD+ supplementation therapy represents a promising but clinically evidence-limited domain. Existing clinical data indicate that NR supplementation modulates inosine signaling in SLE patient-derived monocytes in an NAD+-dependent manner, thereby suppressing IFN signaling pathway activation (303), while evidence for NMN is derived primarily from studies of cutaneous lupus demonstrating NAD+ metabolic dysregulation at cutaneous lesion sites, with reduced NMN and NAD+ concentrations (304). The current evidence base is insufficient to support NAD+ supplementation as standard adjunctive therapy; however, given NAD+’s established roles in immune cell metabolism, DNA repair, and SIRT protein regulation, the prospects for this therapeutic modality are promising as the investigational evidence base continues to expand.

### 4.3 Barriers to the implementation of mitochondria-targeted strategies in SLE

The clinical application of sophisticated mitochondria-targeted therapeutic approaches in SLE remains extremely limited, and the reasons underlying this gap extend well beyond simple investigational lag or technological immaturity.

The primary barrier is the mechanistic complexity of the disease itself. Our understanding of the specific pathways through which mitochondrial dysfunction and mtDNA damage contribute to SLE pathogenesis remains incomplete, and fundamental questions persist regarding the convergent and divergent mechanisms by which mtDNA dysregulation drives inflammation across heterogeneous patient populations, cell types, and disease stages. Without a more precise understanding of disease-specific molecular targets, the development of truly precision therapeutic strategies is inherently constrained.

Technical challenges constitute a second major impediment. Targeted drug development for mitochondrial applications must overcome substantial obstacles related to delivery efficiency, tissue specificity, and bioavailability. MitoQ, as an illustrative case, despite showing encouraging efficacy *in vitro* and in animal models, requires rigorous characterization of human pharmacokinetics, long-term safety profiles, and immune cell-specific enrichment properties before clinical advancement can proceed. Achieving tissue-specific mitochondrial drug delivery in a systemic disease that simultaneously involves multiple immune cell lineages and organ systems represents a formidable challenge that current delivery technologies have not resolved.

A third critical barrier lies in the complexity of clinical trial design and outcome evaluation for this therapeutic class. The extraordinary heterogeneity of SLE, encompassing wide inter-patient variability in clinical manifestations, disease severity, and organ involvement, makes trial design inherently difficult. Mitochondria-targeted therapies may require prolonged treatment periods to demonstrate efficacy, given that they modulate immune responses indirectly through improvements in cellular metabolic homeostasis rather than through direct immunosuppression. Conventional SLE clinical trial endpoints, which predominantly capture short-term disease activity fluctuations, may be inadequate to detect the longitudinal benefits of such interventions. Furthermore, while mtDNA copy number, oxidative damage biomarkers, and mitochondrial functional parameters represent plausible candidate surrogate endpoints, their correlation with clinical outcomes has not been established and standardized measurement methodologies are lacking.

Advanced strategies, including mitochondrial transplantation and gene therapy, face additional disease-specific obstacles. For mitochondrial transplantation, fundamental questions remain unresolved: under what conditions should transplantation be performed, whether single transplantation is sufficient or repeated procedures are required, and which cell populations represent the most appropriate therapeutic targets. Given the likelihood of intrinsic mitochondrial functional deficits or genetic predispositions in SLE patients, autologous transplantation is largely precluded, raising the unresolved question of how allogeneic immunological rejection risks can be adequately mitigated. The logistical requirements of mitochondrial procurement, purification, preservation, quality control, and delivery each present independent challenges, and studies have demonstrated that even optimally preserved mitochondrial preparations undergo substantial degradation within short post-transplantation intervals (305, 306). The ultimate fate of transplanted mitochondria within the inflammatory milieu of SLE, including their viability, integration into host mitochondrial networks, and functional reconstitution, remains poorly characterized, and the prospect of mitochondrial transplantation becoming a mainstream SLE therapeutic modality in the foreseeable future appears remote.

Direct mtDNA gene editing remains in early developmental stages. Although CRISPR-Cas9 technology has been successfully applied to nuclear genome editing and has entered clinical use for genetic disorders such as sickle cell disease (307, 308) and β-thalassemia (309), mtDNA editing faces unique challenges, including the substantial barrier to intramitochondrial delivery of Cas9 protein and guide RNA posed by the double-membrane architecture, the technical requirement to edit a sufficient proportion of the hundreds to thousands of mtDNA copies per cell to achieve therapeutic effects, and the absence of efficient homologous recombination repair in mitochondria that would enable precise sequence correction rather than mere disruption. Compounding these technical limitations, pathogenic mtDNA loci in SLE remain incompletely characterized, and unlike monogenic mitochondrial disorders, mitochondrial dysfunction in SLE predominantly reflects functional dysregulation rather than specific genetic mutations, substantially limiting the conceptual applicability of gene editing approaches.

### 4.4 Future prospects for mtDNA-targeted therapy in SLE

Although the clinical implementation of mitochondria-targeted therapeutics in SLE remains at an early stage, a synthesis of current scientific evidence and emerging technological trends allows for a reasoned delineation of the field’s prospective developmental directions.

In terms of pharmacological innovation, the development of compounds capable of more precisely engaging defined therapeutic targets represents a priority need. Among the most compelling candidates are small-molecule inhibitors of the cGAS-STING pathway and antagonists of TLR9, both of which sit at the convergence point between mtDNA release and downstream inflammatory amplification. Several cGAS inhibitors, including RU.521 and G140, have demonstrated efficacy in preclinical models of autoimmune disease, reducing type I interferon production and ameliorating lupus-like phenotypes (310–312), and their further development toward clinical candidates represents an active area of investigation. At the level of STING, covalent antagonists such as H-151 and C-176 have shown capacity to suppress interferon signaling in murine models of sterile inflammation and are being evaluated for their potential in chronic autoimmune settings (313–315). For TLR9, the therapeutic landscape is particularly nuanced in SLE: hydroxychloroquine, already a cornerstone of SLE management, exerts part of its anti-inflammatory effect through disruption of endosomal TLR signaling, providing indirect clinical validation for this target (316, 317). More selective TLR9 antagonists, including oligonucleotide-based inhibitors such as IMO-8400 (318), are under investigation in related autoimmune conditions and may ultimately prove applicable in SLE subsets with pronounced mtDNA-driven interferon activation. Critically, because cGAS-STING and TLR9 are activated not only by mtDNA but also by nuclear DNA and other endogenous nucleic acid species, their targeted inhibition may offer broader disease-modifying benefits that extend beyond the mitochondrial compartment, making them particularly attractive candidates for integration into SLE therapeutic frameworks.

Combination therapeutic approaches are also likely to prove substantially more effective than monotherapy. The synergistic integration of mitochondria-targeted interventions with established immunosuppressants or biological agents, such as the pairing of NAC or metformin with belimumab or anifrolumab, may generate additive or multiplicative therapeutic benefits that neither agent achieves independently (319, 320). Such combinations are particularly rational in SLE, where multiple pathological loops operate simultaneously, and targeting only one node is unlikely to achieve durable disease control.

From a disease management perspective, lifestyle interventions represent an underappreciated but clinically accessible mitochondria-targeted strategy. Structured exercise protocols have been shown to promote mitochondrial biogenesis and improve mtDNA quality (12), while moderate aerobic exercise and resistance training demonstrably improve symptom burden and quality of life in SLE patients (321, 322). Adherence to specific dietary patterns, most notably the Mediterranean diet, similarly holds potential for modulating mitochondrial function (323, 324), and professionally supervised dietary interventions can further support disease management (325, 326). Comprehensive strategies that integrate pharmacological therapy with structured lifestyle modification may generate additive or synergistic benefits across both metabolic and immunological endpoints.

Technological advances in drug delivery represent another promising avenue. Progress in nanomedicine enables the rational design of cell- and organ-specific mitochondria-targeted delivery platforms, including liposomal formulations and polymeric nanoparticles surface-modified with cellular biomarker recognition elements and mitochondria-targeting peptides or triphenylphosphonium moieties, which in principle can achieve highly efficient and selective intramitochondrial drug delivery (327, 328).

The integration of precision medicine approaches with mtDNA biology offers perhaps the most consequential long-term prospect for this field. Drawing on the biomarker parameters and mechanistic evidence discussed above, we propose that SLE patients might provisionally be stratified into two mtDNA-defined pathological subtypes with distinct therapeutic implications. The first, which we term the high mtDNA release subtype, is characterized by markedly elevated circulating cell-free mtDNA, a strong type I interferon gene signature, and a predilection for lupus nephritis and endothelial injury; such patients might derive particular benefit from cGAS-STING inhibition, TLR9 antagonism, and strategies to restore mitophagy or reduce NET formation. The second, the oxidative mtDNA damage subtype, would be defined by high leukocyte 8-OHdG burden, prominent NLRP3 inflammasome activation, and a clinical profile skewed toward cardiovascular involvement and thrombotic risk, and might respond preferentially to mitochondria-targeted antioxidants, NAD+ repletion, or anti-IL-1β strategies. Anti-TFAM antibody positivity may delineate a thrombosis-prone subset within the latter group, for whom anticoagulation rather than intensified immunosuppression could warrant consideration.

**Figure 4:**
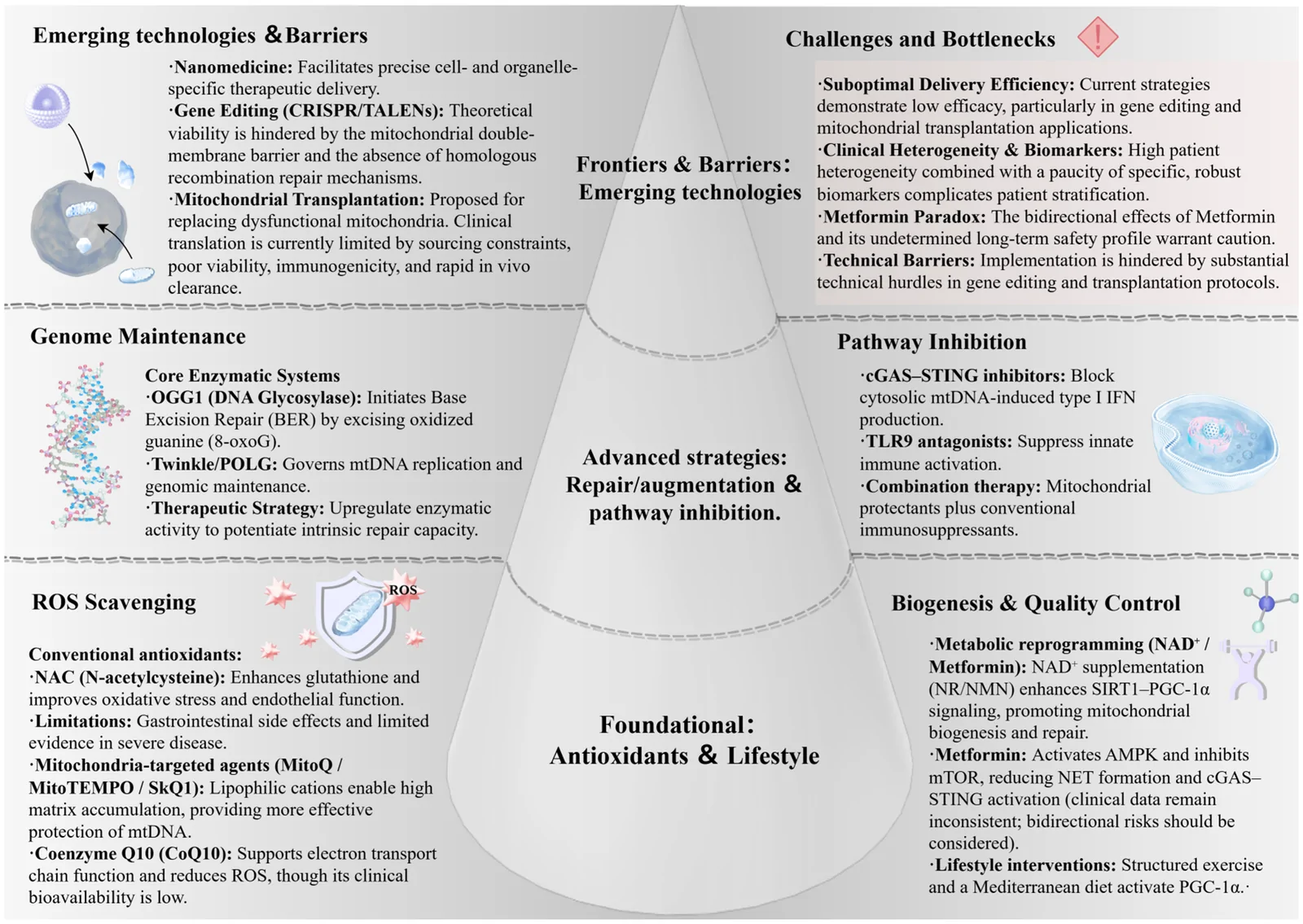
Multi-dimensional therapeutic strategies targeting mtDNA in SLE. In response to the diverse mtDNA-associated pathological processes and inflammatory cascades that characterize SLE, therapeutic approaches are evolving from single-agent antioxidant interventions toward integrated strategies that simultaneously target metabolic, genomic, and immunological dimensions of disease. Mitochondria-directed antioxidants, including MitoQ and coenzyme Q10, exploit lipophilic cationic conjugation to achieve preferential accumulation within the mitochondrial matrix, enabling direct attenuation of oxidative mtDNA damage in a manner that conventional antioxidants, which lack adequate mitochondrial enrichment, cannot accomplish. Metabolic and biogenesis-promoting strategies employ NAD+ precursors, metformin-mediated AMPK activation, and lifestyle interventions including structured exercise and dietary modification to activate the PGC-1α/SIRT1 transcriptional axis, thereby reinforcing mitochondrial quality control networks and preserving mtDNA structural integrity. Genomic integrity-focused approaches aim to enhance OGG1-mediated base excision repair and optimize the replicative fidelity of Polγ and Twinkle. While mitochondrial transplantation and genome editing technologies remain in early investigational stages, constrained by challenges including suboptimal intramitochondrial delivery and allogeneic immunological barriers, downstream therapeutic strategies targeting the cGAS-STING and TLR9 inflammatory axes, combination regimens integrating mitochondria-targeted agents with established immunosuppressants, and nanoparticle-based delivery platforms informed by artificial intelligence-driven patient stratification collectively represent a promising therapeutic landscape for disrupting mtDNA-driven pathological circuits and advancing individualized treatment in SLE.

We emphasize that this framework, summarized in **Table 3**, is a hypothesis rather than a validated classification. It is derived from associations observed across separate cohorts rather than from any single study in which these parameters were measured together, and the two subtypes are unlikely to be mutually exclusive in individual patients. Its value lies in generating testable predictions for biomarker-stratified trial design, and prospective validation in adequately powered cohorts would be required before any clinical application. Machine learning approaches to predicting individual treatment response may eventually refine such a framework, but only once the underlying associations have been independently confirmed (**Tables 3**, **4**; **Figure 4**).

**Table 3:** Proposed mtDNA-based patient stratification framework for therapeutic decision-making in SLE.

Feature	High mtDNA release subtype	Oxidative mtDNA damage subtype
Key biomarkers	Elevated circulating cfDNA; abnormal cfDNA fragment profile	Elevated leukocyte 8-OHdG; high anti-mtDNA IgG
Immune signature	Strong type I IFN gene signature; elevated anti-mtDNA antibodies	NLRP3 inflammasome activation; elevated IL-1β
Clinical predilection	Lupus nephritis; endothelial dysfunction; vasculitis	Cardiovascular disease; thrombosis; antiphospholipid syndrome
Additional marker	High NET-associated MPO-DNA complexes; LDG expansion	Anti-TFAM antibody positivity; IFN-λ elevation
Preferred therapeutic targets	cGAS-STING inhibition; TLR9 antagonism; mitophagy restoration; NET suppression	Mitochondria-targeted antioxidants; NAD^+^ repletion; anti-IL-1β; anticoagulation (if anti-TFAM positive)
Potential combination strategy	Metformin or NAC + anifrolumab	Coenzyme Q10 or MitoQ + IL-1 antagonist
Validation status	Provisional; requires prospective cohort validation	Provisional; requires prospective cohort validation

**Table 4:** Current status and prospects of mtDNA-targeted therapeutic strategies in systemic lupus erythematosus.

Therapeutic category	Representative agents/methods	Mechanism of action	Clinical evidence	Current status	Major challenges
Mitochondria-Targeted Antioxidants	Coenzyme Q10	ETC component; ROS scavenging	Improved renal and vascular endothelial function in animal models	Preclinical research	Poor bioavailability; lack of clinical trial data
	MitoQ	Lipophilic cation–CoQ10 conjugate targeting mitochondrial matrix	Good safety profile in neurodegenerative and cardiovascular diseases	Not tested in SLE patients	Absence of SLE clinical evidence; requires safety data accumulation
	MitoTEMPO/SkQ1	Lipophilic cation-conjugated antioxidants	Basic research stage	Preclinical research	Clinical translation distant
Conventional Antioxidants	N-Acetylcysteine (NAC)	Glutathione precursor; elevates intracellular GSH levels	Reduced SLEDAI scores; improved T cell and endothelial function	Preliminary clinical trial success	Small sample sizes; gastrointestinal adverse events; lack of long-term follow-up
	Vitamin E/C	Traditional free radical scavengers	Low mitochondrial enrichment efficiency	Limited efficacy	Poor mitochondrial targeting
Metabolic Modulators	Metformin	AMPK activation; mTOR inhibition; mitochondrial optimization	Conflicting RCT outcomes	Mixed clinical evidence	Unclear patient stratification; bidirectional effects
	Nicotinamide Riboside (NR)	Elevates NAD+; activates SIRT1; promotes biogenesis	Influences IFN signaling in SLE monocytes	Preliminary evidence	Very limited clinical evidence
	Nicotinamide Mononucleotide (NMN)	NAD+ precursor; regulates DNA repair	Observed NAD+ dysregulation in cutaneous lupus	Preliminary evidence	Lack of systemic SLE studies
Biogenesis Promoters	Exercise Training	PGC-1α activation	Improved symptoms and quality of life	Lifestyle intervention	Compliance; individualized protocols required
	Resveratrol	Activates PGC-1α/SIRT1 axis	Animal model evidence	Preclinical research	Insufficient clinical translation
Signaling Pathway Inhibitors	cGAS-STING inhibitors	Block mtDNA-induced type I IFN	Theoretical target	Drug development stage	No clinical candidates yet
	TLR9 antagonists	Block CpG-mtDNA recognition	Theoretical target	Drug development stage	Requires precise patient stratification
Advanced Biotechnology	Mitochondrial transplantation	Replacement of dysfunctional mitochondria	Success in myocardial ischemia, Parkinson’s disease	Proof-of-concept	Delivery, immune rejection, systemic unsuitability
	Gene editing (CRISPR)	Correction of mtDNA mutations	Basic research	Early stage	Technical barriers; mtDNA multicopy nature
Combination Therapy	Mediterranean diet + medications	Multi-pathway synergy	Observational benefit	Exploratory	Needs controlled trials
Nanomedicine Platforms	Targeted mitochondrial delivery	Cell/organ-specific targeting	Preclinical	Early development	Requires clinical translation

## 5 Conclusion

It is now increasingly clear that mtDNA occupies a central and mechanistically active role in the pathogenesis of SLE, far beyond its traditional recognition as a passive marker of mitochondrial dysfunction. Endowed with its bacterial evolutionary origins, heightened susceptibility to oxidative mutagenesis, and dynamically regulated nucleoid architecture, mtDNA is implicated in disease initiation, amplification of innate and adaptive immune responses, and the pattern of organ-specific injury across the disease course. The paradoxical coexistence of intracellular mtDNA depletion and extracellular accumulation that characterizes SLE not only provides a sensitive reflection of disease activity but also positions mtDNA as a molecular bridge connecting mitochondrial dysfunction to systemic immune dysregulation. The release of mtDNA from its protected intramitochondrial environment into the cytosol and extracellular space proceeds through a series of mechanistically defined steps, encompassing ultrastructural mitochondrial remodeling, TFAM-mediated nucleoid disassembly, pathological mPTP opening, and failure of autophagic surveillance. Once liberated, mtDNA engages innate immune sensors including cGAS-STING, TLR9, and inflammasome platforms, initiating inflammatory circuits that appear to coordinate immune dysregulation across multiple cell lineages and contribute to tissue-specific injury.

The therapeutic potential of targeting mtDNA-centered pathways in SLE has been compellingly established at the conceptual level, yet the translation of this potential into clinical practice remains a formidable challenge. Early clinical evidence supporting NAC and metformin requires validation through larger, more rigorously designed trials, while more advanced therapeutic strategies, including MitoQ, NAD+ repletion, mitochondrial transplantation, and gene editing, remain at investigational stages and await the mechanistic and technological breakthroughs necessary for clinical application. Progress toward bedside implementation will require deeper mechanistic understanding of mtDNA pathobiology in SLE, more precise patient stratification frameworks that identify subpopulations most likely to benefit from mitochondria-targeted interventions, and the development of innovative therapeutic platforms capable of achieving selective delivery to pathological sites within a complex systemic disease. Realizing these advances will be essential for mtDNA-targeted strategies to fulfill their promise as meaningful contributors to the comprehensive management of SLE.
